# Pedoclimatic Conditions Influence the Morphological, Phytochemical and Biological Features of *Mentha pulegium* L.

**DOI:** 10.3390/plants12010024

**Published:** 2022-12-21

**Authors:** Laura Cornara, Federica Sgrò, Francesco Maria Raimondo, Mariarosaria Ingegneri, Luca Mastracci, Valeria D’Angelo, Maria Paola Germanò, Domenico Trombetta, Antonella Smeriglio

**Affiliations:** 1 Department of Earth, Environment and Life Sciences, University of Genova, C.so Europa 26, 16132 Genova, Italy; 2 Foundation Prof. Antonio Imbesi, University of Messina, Piazza Pugliatti 1, 98122 Messina, Italy; 3 PLANTA/Autonomous Center for Research, Documentation and Training, Via Serraglio Vecchio 28, 90123 Palermo, Italy; 4 Department of Chemical, Biological, Pharmaceutical and Environmental Sciences, University of Messina, Viale Ferdinando Stagno d’Alcontres 31, 98166 Messina, Italy; 5 Pathology Unit, Department of Surgical and Diagnostic Sciences (DISC), University of Genova, 16132 Genova, Italy; 6 Ospedale Policlinico San Martino, Largo Rosanna Benzi 10, 16125 Genova, Italy

**Keywords:** *Mentha pulegium* L., pedoclimatic growth conditions, micromorphology, polyphenols, phytochemical profile, antioxidant activity, anti-inflammatory activity, antiangiogenic properties

## Abstract

In this study, *Mentha pulegium* leaves and flowers harvested in three different Sicilian areas were investigated from a micromorphological, phytochemical and biological point of view. Light and scanning electron microscopy showed the presence of spherocrystalline masses of diosmin both in the leaf epidermal cells and in thin flower petals. Two different chemotypes were identified (I, kaempferide/rosmarinic acid; II, jaceidin isomer A). Phytochemical screening identified plant from collection site II as the richest in total phenolics (16.74 g GAE/100 g DE) and that from collection site I as the richest in flavonoids (46.56 g RE/100 g DE). Seventy-seven metabolites were identified both in flower and leaf extracts. Plant from site II showed the best antioxidant (0.90–83.72 µg/mL) and anti-inflammatory (27.44–196.31 µg/mL) activity expressed as half-maximal inhibitory concentration (IC_50_) evaluated by DPPH, TEAC, FRAP, ORAC, BSA denaturation and protease inhibition assays. These data were also corroborated by in vitro cell-based assays on lymphocytes and erythrocytes. Moreover, plant of site II showed the best antiangiogenic properties (IC_50_ 33.43–33.60 µg/mL) in vivo on a chick chorioallantoic membrane. In conclusion, pedoclimatic conditions influence the chemotype and the biological activity of *M. pulegium*, with chemotype I showing the most promising biological properties.

## 1. Introduction

The genus *Mentha*, belonging to the *Lamiaceae* family, has a very complicated taxonomy, including approximately 42 species and 15 hybrids, with hundreds of subspecies and cultivars widespread worldwide [1]. *Mentha* essential oils (EOs) and extracts are widely used as natural ingredients in herbal cosmetics and in different pharmaceutical preparations [2] as antipyretic, spasmodic/antispasmodic, bronchodilator, and carminative agents [3,4]. In addition, phytochemicals derived from mint have shown cytotoxic effects on different kinds of human cancer, such as cervix, lung, breast, and many other cancers [5,6].

Among Italian and Sicilian taxa, one of the most diffused is *Mentha pulegium* L., commonly known worldwide as mosquito plant, pennyroyal mint, pennyrile, pudding grass or squaw mint [1]. It is an aromatic and tomentose perennial herb, showing an indumentum characterized by non-glandular and glandular trichomes of both capitate and peltate kinds typical of the *Lamiaceae* family [7]. This species, which is widespread in Europe, the Middle East and North Africa, is able to grow under different environmental conditions [8].

Traditionally, this plant was used in ancient times in Greek, Roman and Medieval cultures as a digestive, emmenagogue, antitussive, antiseptic, and abortifacient [9,10]. Recent studies have shown that *M. pulegium* can be particularly indicated as an adjuvant in chronic diseases such as cancer, diabetes and neurodegenerative pathologies [11], owing to its antioxidant [12], antiviral [13] and antibacterial/antibiofilm properties [14,15].

With a strong scent similar to that of spearmint, *M. pulegium* has been used for centuries as herbal tea for cold relief, coughs, kidney problems and headaches, as well as a food preservative/flavoring and insect repellent [16,17]. *M. pulegium* tea and leaf extracts have been used without reported side effects. On the contrary, *M. pulegium* EO is highly toxic, and even small oral doses (≥15 mL) can cause syncope, seizures, coma, cardiopulmonary collapse, acute liver injury, renal insufficiency and multiorgan failure [18]. For these reasons, it is not indicated for medical purposes but only in aromatherapy as a bath additive and as a pesticide. Recently, we studied the phytotoxic activity of *M. pulegium* EO, highlighting one of the possible mechanisms of action and showing its low ecotoxicity, which would make it useful as a bioherbicide [19].

The toxicity of *M. pulegium* EO has been ascribed mainly to its pulegone content [17,20]. However, in our recent study, we showed that the volatile phytochemical profile of *M. pulegium* is strongly influenced by pedoclimatic growth conditions, leading to different chemotypes, of which the most common are pulegone/isomenthone and piperitone/isomenthone. It seems that more than the stress of altitude, salinity stress shifts the metabolic pathway towards the biosynthesis of pulegone [19]. However, other than the volatile fraction rich in alcohols, ketones, esters, ethers and oxides, *M. pulegium* is also a rich source of polyphenols [1]. Hydroxycinnamic acid derivatives including rosmarinic and salvianolic acid conjugates, as well as flavonoids such as quercetin, isorhamnetin, naringenin, and gallocatechin derivatives, represent the most abundant compounds. These bioactive components seem to play a pivotal role in the biological properties ascribed to the plant complex obtained from *M. pulegium* [21].

It has been shown that pedoclimatic conditions can also influence the morphoanatomical and polyphenolic profile of plants and, consequently, the biological activity of their extracts [22,23]. However, only a few studies have been conducted to date on *M. pulegium* in this regard.

Accordingly, the aim of the present study was to investigate, by a pharmacognostic approach, wild populations of *M. pulegium* growing in different areas of the Sicily region. Therefore, we analyzed the influence of different pedoclimatic conditions on macro- and micromorphological features of leaves and flowers (MPL and MPF, respectively), as well as the phytochemical and biological properties of their hydroalcoholic extracts.

## 2. Results

### 2.1. Micromorphological Analyses

Both leaves and flowers of *M. pulegium* were characterized by the presence of non-glandular and glandular trichomes of different types, as commonly found in the Lamiaceae family.

Micromorphological analysis highlighted the presence of spherocrystals attributable to the flavonoid diosmin both in the leaves and in the flowers of all three samples (MPI, MPII and MPIII). The distribution of the crystals appeared to differ slightly in leaves and flowers of plants growing in different locations, although microscopic analysis does not allow for clear quantification of differences in diosmin content between the samples. A comparison between microscopic analysis and diosmin chemical determination is shown in Table 1.

**Figure 1:**
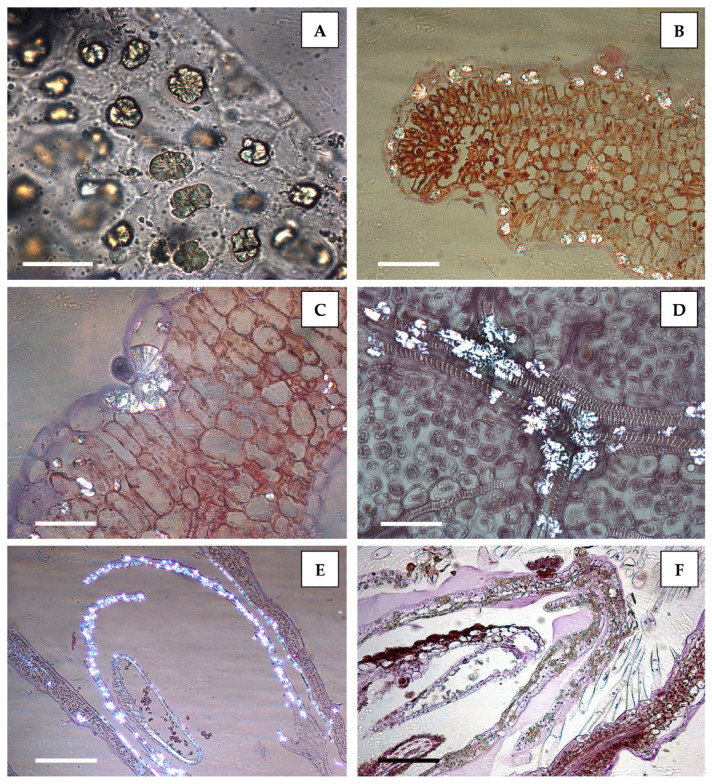
Spherocrystalline masses of diosmin were abundant in the leaf epidermal cells (**A**,**B**), sometimes crowded at the base of trichomes (**C**), as well as along the leaf veins (**D**). These crystals were also visible inside the thin petals of the flowers (**E**,**F**). A: Leaf peelings mounted in water, where diosmin spherocrystals appear unstained. (**B**–**F**): Paraffin-embedded samples and semithin sections stained with hematoxylin–eosin. Cross section (**B**,**C**) and transdermal section (**D**) of a leaf; longitudinal section of a flower (**E**,**F**). Aggregates of diosmin crystals were birefringent under polarized light in both leaves and flowers (**B**–**E**) but remained unstained when treated with hematoxylin–eosin staining (**F**). Bars: A = 50 µm; B = 100 µm; C = 50 µm; D = 50 µm; E = 100 µm; F = 100 µm.

Based on light microscopy (LM), the crystalline masses appeared to be very abundant in the leaf epidermal cells (Figure 1A,B), sometimes crowded around the base of trichomes (e.g., Figure 1C, around the base of a capitate glandular trichome) and sometimes also found within non-glandular trichomes (not shown).

In addition, crystals were frequently abundant along the leaf veins (Figure 1D). They were also present in the flowers, particularly inside the thin flower petals (Figure 1E,F). Diosmin crystals were birefringent under polarized light both in handmade sections or peelings (not shown) and in semithin sections (Figure 1B–E).

**Figure 2:**
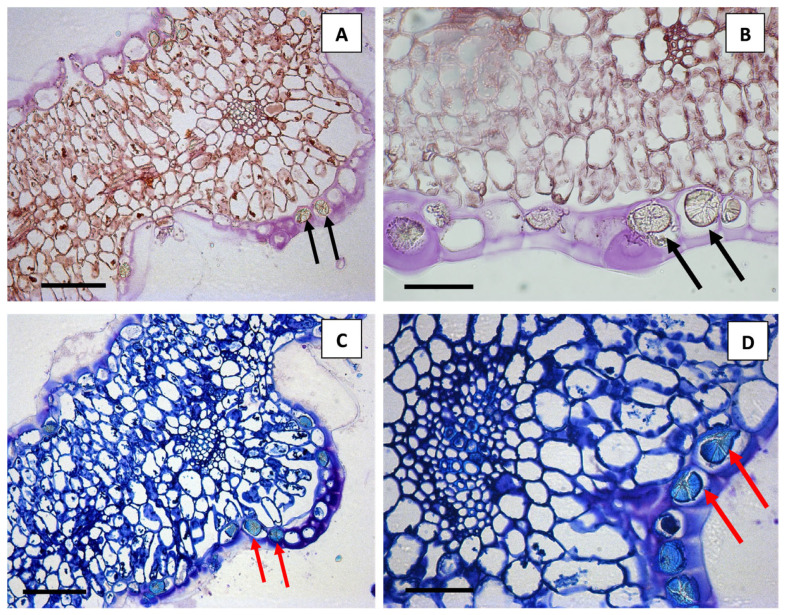
Leaf semithin transversal sections ((**A**,**B**) hematoxylin–eosin staining; (**C**,**D**) TBO staining). Diosmin spherocrystals appeared unstained with hematoxylin–eosin ((**A**,**B**) black arrows) but showed typical bright-blue staining of phenolic compounds with TBO ((**C**,**D**) red arrows). Scale bars: A = 100 µm; B = 50 µm; C = 100 µm; D = 50 µm.

The crystals appeared unstained in handmade sections or peelings, mounted in water (Figure 1A) and in semithin sections stained with hematoxylin–eosin (Figure 1F and Figure 2A,B, black arrows).

On the contrary, TBO stained these spherocrystals with the typical bright-blue staining of phenolics (Figure 2C,D), confirming their flavonoid composition.

**Figure 3:**
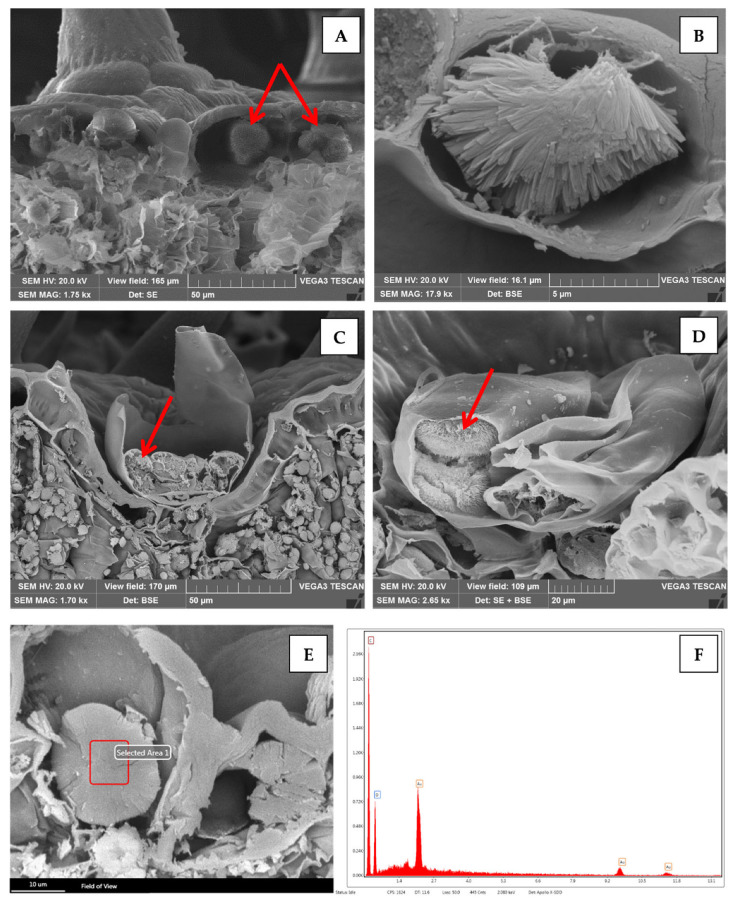
Transversal section of leaf analyzed by SEM (**A**–**D**) and SEM-EDX (**E**,**F**). Diosmin spherocrystal aggregates are visible within epidermal cells ((**A**) red arrows); a more detailed morphological characterization of a spherocrystal within a single epidermal cell is shown at higher magnification (**B**). Diosmin spherocrystals were also present in the secretory cells of the peltate glandular trichomes ((**C**,**D**) red arrow). SEM-EDX analysis confirmed the absence of mineral elements in the crystal composition (**E**,**F**).

Scanning electron microscopy (SEM) analysis provided a more detailed morphological characterization of these spherocrystals (Figure 3A, red arrows, and Figure 3B).

In addition, in transversal sections of the leaf, these crystals were also found within the secretory cells of the peltate glandular trichomes (Figure 3C,D, red arrow). This site of localization of diosmin crystals within plant tissues, to the best of our knowledge, is revealed here by micromorphology analysis for the first time in *M. pulegium*. The elemental composition of these organic crystals was confirmed by SEM X-ray energy-dispersive (SEM-EDX) analysis, showing the presence of carbon and oxygen and assessing the absence of mineral elements (Figure 3E,F). The peaks marked “Au” in Figure 3F correspond to the gold sputter coating of the sample.

### 2.2. Phytochemical Analyses

A preliminary phytochemical screening was carried out to quantify total phenolic, flavonoid and flavan-3-ol contents in *M. pulegium* leaf and flower extracts (Table 2). MPLE II showed the best total phenolic content, followed by MPLE III and MPLE I. On the contrary, MPLE I showed the best flavonoid and flavan-3-ol contents, followed by MPLE III and MPLE II. Among the flower extracts, MPFE I showed the best total phenolic content, followed by MPFE II and MPFE III. MPFE III showed the highest content of flavonoids, followed by MPFE I and MPFE II. Furthermore, MPFE III is the richest in flavan-3-ols, whereas MPFE I and MPFE II did not show any statistically significant difference. Surprisingly, especially in the case of MPLE I and MPFE III, the total phenolic content recorded by the Folin–Ciocalteu assay is much lower than the total flavonoid content. This can be explained by the fact that this colorimetric test is highly dependent on the phytochemical profile, which can differ in terms of the content of phenolic types and the amounts of specific compounds [24,25]. Furthermore, gallic acid, although it is the most widely used reference compound, is not the compound that best represents the reaction behavior of the samples under study, which are characterized by polymeric and condensed phenolic compounds [26]. Because different phenols react to different degrees, the expression of results as gallic acid equivalents is arbitrary, but necessary to compare the results with the available literature data.

**Figure 4:**
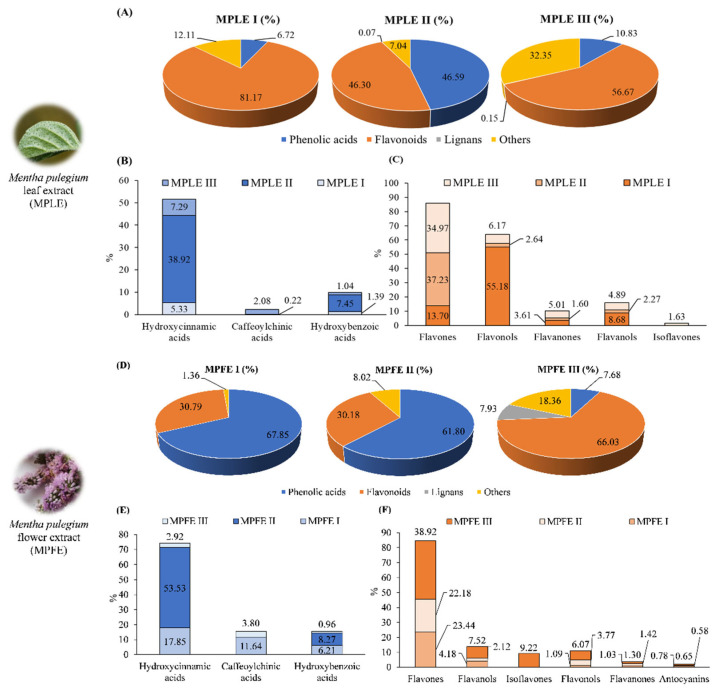
Comparison of the mean percentage (%) distribution of the different classes of polyphenols identified in the extracts of leaves (**A**–**C**) and flowers (**D**–**F**) of *M. pulegium* (MPLE and MPFE, respectively). Results are expressed as mean area percentage (%) based on LC-DAD data acquired at 280 nm with respect to the total area of identified and unidentified (unknown) compounds.

These preliminary data were confirmed by LC-DAD-ESI-MS analysis. By grouping all the identified polyphenols into classes and comparing the extracts obtained from the leaves and flowers from the three collection sites (I, II and III), it is immediately clear that MPLE II is the richest in phenolic acids (mainly hydroxycinnamic acids), followed by MPLE III (mainly hydroxycinnamic acids) and MPLE I (Figure 4).

The same result was found for the abundances of flavonoids. According to previously observations in colorimetric tests (Table 2), MPLE I is the richest in flavonoids and flavan-3-ols, followed by MPLE III and MPLE II. In addition, it is also interesting to observe the percentage distribution of the other classes of flavonoids among the various investigated extracts. In particular, MPLE I showed the highest content of flavonols, whereas MPLE II and MPLE III showed the highest content of flavones and flavanones, respectively (Figure 4C). Regarding flower extracts, in this case, the results of the LC-DAD-ESI-MS analysis are in agreement with the phytochemical screening. MPFE I is the richest in phenolic acids (mainly hydroxycinnamic and hydroxybenzoic acids), followed by MPFE II (mainly hydroxycinnamic acids) and MPFE I (Figure 4E). MPFE III is the richest in flavonoids and flavan-3-ols, followed by MPFE I, and MPFE II (Figure 4F). It is very interesting to note that, in contrast to observations in leaf extracts, in flower extracts, regardless of the collection site, the most abundant class of flavonoids is always that of flavones (Figure 4F).

In a more detailed analysis of the phytochemical profile elucidated through LC-DAD-ESI-MS analysis, 112 compounds were detected, of which 91 were identified (Figures S1–S6). The results reported in Table 3 are expressed as mean area percentage ± the standard deviation of nine independent analyses in triplicate (*n* = 3) with respect to the total area of identified and unidentified (unknown) compounds. The phytochemical profile of the leaf extracts, as well as that of the flower extracts, from the three different locations (I, II and III), despite some similarities, appears significantly different (*p* < 0.001). The most abundant compounds (reported in bold in Table 3) identified in MPLE I are kaempferide (52.81%), galloyl galactaric acid (8.71%), gallocatechin (8.31%), jaceidin isomer A (4.84%), luteolin (3.65%) and diosmin (3.61%). MPLE II showed the best content of rosmarinic acid (19.16%), followed by jaceidin isomer A (14.47%), salvianolic acid I (8.41%), isorhamnetin 3 -glucuronide (8.04%), dihydroxyphenyl galloyl-β-D-glucopyranoside (7.14%) and salvianolic acid E (6.08%) (Table 3). Finally, the most abundant compounds identified in MPLE III are jaceidin isomer A (23.63%), riboflavin 5′-(dihydrogen phosphate) (19.23%), luteolin-7-*O*-rutinoside (5.36%), epigallocatechin caffeate (4.0%), dimethyl epigallocatechin gallate (3.52%) and diosmin (3.33%) (Table 3).

Regarding flower extracts, rosmarinic acid (24.31% and 25.52%, respectively) and jaceidin isomer A (15.73% and 13.45%, respectively) were the most abundant compounds identified in both MPFE I and MPFE II (Table 3). The most abundant compounds of MPFE I are 3,4-dicaffeoylquinic acid (11.64%), salvianolic acid I (6.82%), dihydroxyphenyl galloyl-β-D-glucopyranoside (6.21%) and salvianolic acid C (6.04%) (Table 3). On the contrary, MPFE II showed, among other abundant compounds, salvianolic acid I (8.76%), dihydroxyphenyl galloyl-β-D-glucopyranoside (8.27%), salvianolic acid B (6.85%) and isosalvianolic acid B (6.41%) (Table 3).

MPFE III showed a completely different phytochemical profile with respect to the previous ones, with jaceidin isomer A as the most abundant compound (30.79%), followed by malonyl-daidzin (8.3%), riboflavin 5′-(dihydrogen phosphate) (7.31%), todolactol A (6.71%), epitheaflagallin 3-*O*-gallate (5.35%) and kaempferol-*O*-glucuronide (5.28%) (Table 3).

**Figure 5:**
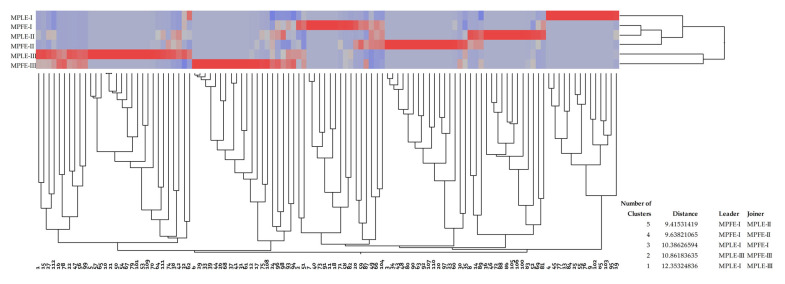
Agglomerative hierarchical clustering analysis of MPLE and MPFE phytochemical profile.

The dendrogram depicted in Figure 5 shows the results of the agglomerative hierarchical clustering analyses performed on the 112 compounds identified and unidentified in MPLE and MPFE. As can be seen from the figure and the respective table, which shows the distances between the different groups, five clusters were identified (Figure 5). Further analyses, such as two-way clustering, showed that some components were mainly present in the extract from one sampling site rather than that of another site. Using a color map, it was possible to identify where the content of a specific constituent was higher (shades of red that darken in proportion to the abundance of the constituent), lower or absent (gray to blue, respectively) (Figure 5).

Thanks to this analysis, two different chemotypes (I and II) can be identified according to the sampling site: kaempferide/rosmarinic acid (collection sites I and II) and jaceidin isomer A (collection site III).

### 2.3. Biological Properties

In this study, the antioxidant and anti-inflammatory properties of MPLE and MPFE were investigated by a two-step approach: a first screening by means of in vitro spectrophotometric and spectrofluorimetric cell-free tests based on different environments and reaction mechanisms and a subsequent screening by means of in vitro cell-based assays.

**Figure 6:**
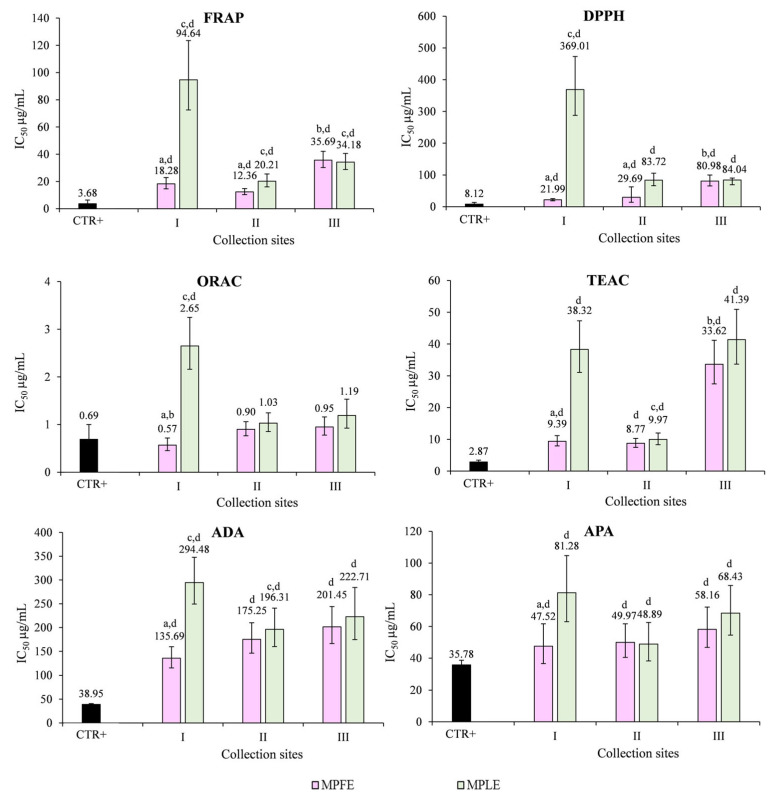
Antioxidant and anti-inflammatory properties of leaf and flower extracts of *M. pulegium* (MPLE and MPFE, respectively) investigated by in vitro cell-free assays. FRAP, ferric-reducing antioxidant power; DPPH, scavenging activity against 2,2-diphenyl-1-picrylhydrazyl radical; ORAC, oxygen radical absorbance capacity; TEAC, trolox equivalent antioxidant capacity; ADA, albumin denaturation assay; APA, antiprotease activity. ^a^ *p* < 0.001 vs. respective MPLE; ^b^ *p* < 0.001 vs. other MPFE; ^c^ *p* < 0.001 vs. other MPLE; ^d^ *p* < 0.001 vs. CTR+ (trolox and diclofenac sodium for antioxidant and anti-inflammatory assays, respectively).

In Figure 6, results of in vitro cell-free assays are depicted and expressed as half-maximal inhibitory concentration (IC_50_, µg/mL) ± confidence limits (C.L.) at 95% able to scavenge the radical activity (FRAP, DPPH, TEAC and ORAC assays) and to inhibit protein denaturation (ADA) and protease activity (APA).

As can be seen from the graph bars, the extracts exhibit superimposable behavior in all the tests performed, except for the FRAP and TEAC assays (Figure 6). The flower extracts show the best and statistically significant (*p* < 0.001 vs. MPLE) antioxidant and anti-inflammatory activity with MPFE I, which shows the lowest IC_50_ values and, consequently, the strongest activity, followed by MPFE II and MPFE III. On the contrary, MPLE II shows the best antioxidant and ant-inflammatory activity, followed by MPLE III and MPLE I (Figure 6). In the FRAP assay, the following order of potency was recorded: MPFE II > MPFE I > MPFE III and MPLE II > MPLE III > MPLE I. In the TEAC assay, the following order of potency was recorded: MPFE II > MPFE I > MPFE III and MPLE II > MPLE I > MPLE III (Figure 6).

**Figure 7:**
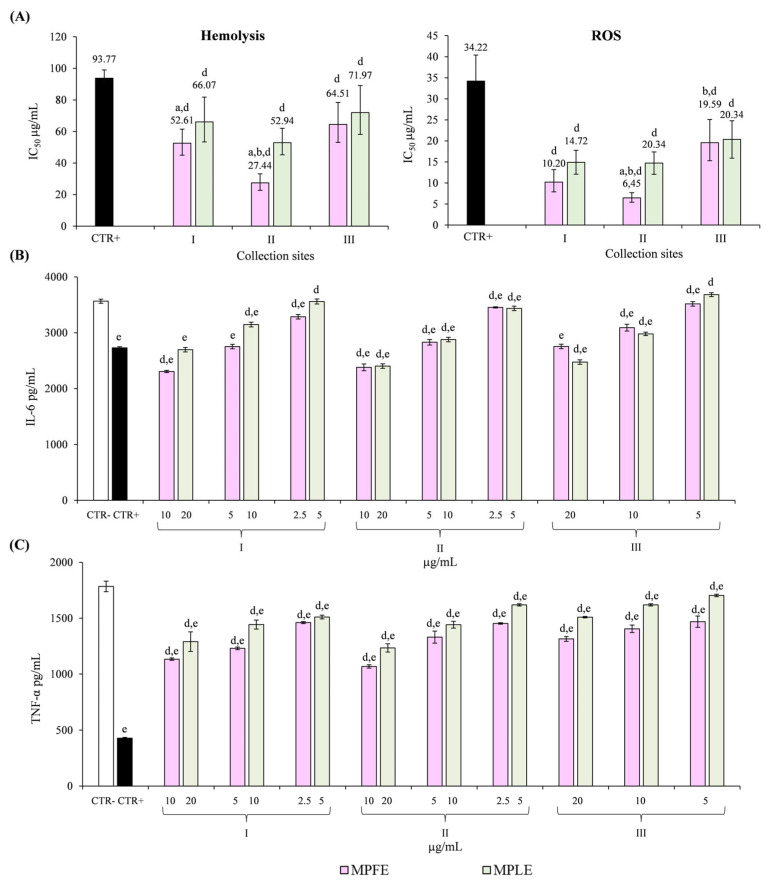
Antioxidant and anti-inflammatory activity of leaf and flower extracts of *M. pulegium* (MPLE and MPFE, respectively) investigated by in vitro cell-based assays carried out on erythrocytes (**A**) and peripheral blood mononuclear cell (PBMC, panel (**B**,**C**)). (**A**) Heat-induced hemolysis, diclofenac sodium (50.0–200.0 µg/mL) was used as positive control (CTR+); free-radical scavenging activity against intracellular ROS, trolox was used as positive control (CTR+); (**B**,**C**) IL-6 and TNF-α release by PBMC after 10 ng/mL LPS-induced inflammation; diclofenac sodium 50 μg/mL was used as positive control (CTR+), whereas cell medium culture containing 0.1% DMSO was used as negative control (CTR-). ^a^ *p* < 0.001 vs. respective MPLE; ^b^ *p* < 0.001 vs. other MPFE; ^c^ *p* < 0.001 vs. other MPLE ^d^ *p* < 0.001 vs. CTR+; ^e^ *p* < 0.001 vs. CTR-.

The same trend observed in the TEAC assay was recorded also in hemolysis and ROS assays carried out on erythrocytes (Figure 7A). In this case, MPFE II and MPLE II showed the strongest activity, followed by MPFE I, MPLE I, MPFE III and MPLE III, confirming previous observations on in vitro cell-free assays. Moreover, it is interesting to note that all the extracts showed significantly (*p* < 0.001) greater activity than the reference standards (CTR+, diclofenac sodium and trolox in hemolysis and ROS assays, respectively) (Figure 7A).

To confirm the anti-inflammatory activity of *M. pulegium* extracts, in vitro cell-based assays were carried out on peripheral blood mononuclear cells (PBMCs) pre-treated with the extracts under examination and stimulated with 10 ng/mL LPS. Results, referring to IL-6 and TNF-α release, two well-known inflammatory markers, are depicted in Figure 7B,C, respectively. Both MPFE and MPLE decreased IL-6 and TNF-α release in a statistically significant (*p* < 0.001) and concentration-dependent manner with respect to the negative control (CTR-), with MPFE always showing the best anti-inflammatory activity. Moreover, the highest concentrations of MPFE and MPLE also showed stronger anti-inflammatory activity with respect to the positive control (CTR+, diclofenac sodium 50 μg/mL), except for MPLE I and MPFE III against IL-6 (Figure 7B) and MPFE III against TNF-α (Figure 7C).

Given the strong anti-inflammatory effect of the extracts under examination and the close correlation between angiogenesis and inflammation, antiangiogenic activity was also investigated in the present study using an in vivo model, the chick chorioallantoic membrane (CAM) assay, which, given its simple, quick and inexpensive features, represents the gold standard for the evaluation of this parameter. This test is based on the ability of the extracts under examination to decrease the number of blood vessel branch points in a standardized area, which was evaluated through the acquisition of pictures using a stereomicroscope. In addition to the qualitative and semiquantitative evaluation obtained by processing the images with appropriate software, it is very useful to quantify the hemoglobin content of the CAM treated with the extracts under examination because this parameter directly correlates with the number and thickness of blood vessels.

**Figure 8:**
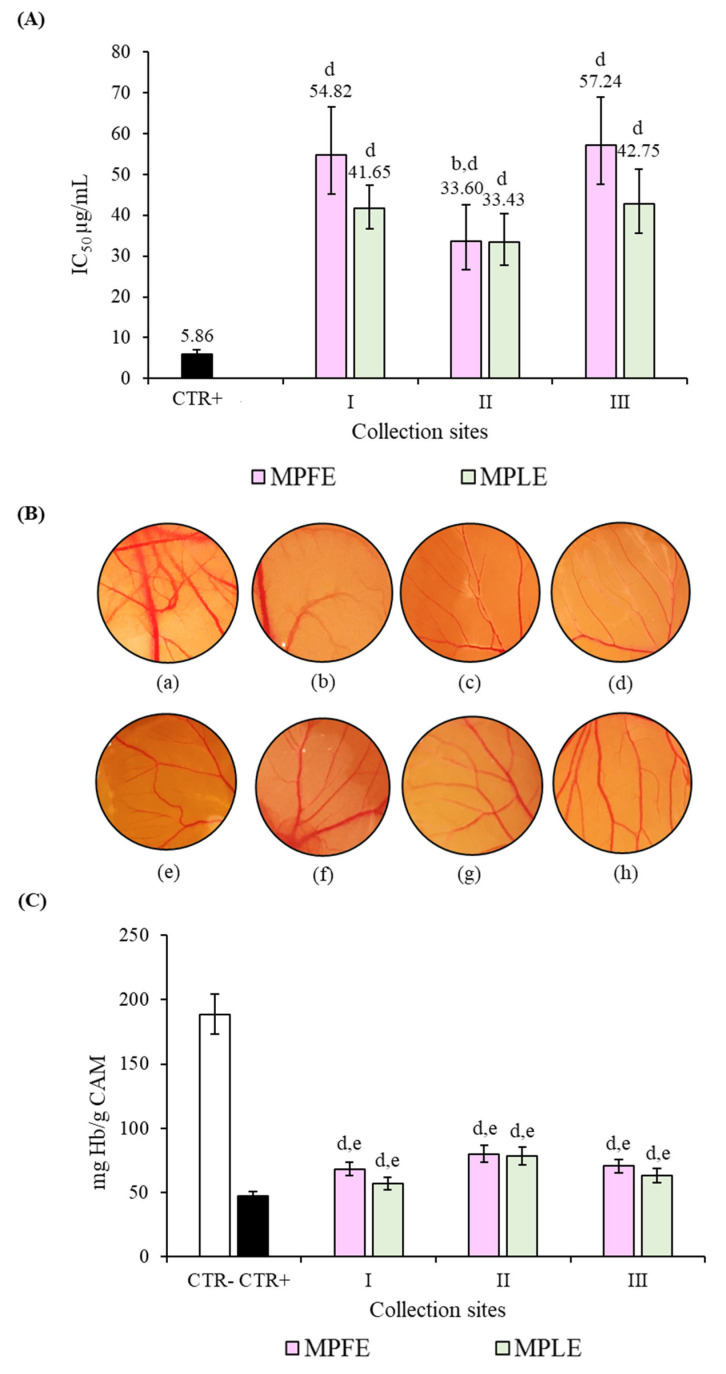
Antiangiogenic activity of leaf and flower extracts of *M. pulegium* (MPLE and MPFE, respectively) investigated by chick chorioallantoic membrane (CAM) assay. (**A**) Antiangiogenic activity expressed as half-maximal inhibitory concentration (IC_50_) of vascular density with respective confident limits (C.L.) at 95%. (**B**) Representative pictures of the CAM treated with CTR- ((**a**) ethanol/water 70:30); CTR+ ((**b**) retinoic acid 10 µg/mL); MPLE I ((**c**) 80 µg/mL), MPLE II ((**d**) 40 µg/mL), MPLE III ((**e**) 80 µg/mL), MPFE I ((**f**) 80 µg/mL), MPFE II ((**g**) 40 µg/mL) and MPFE III ((**h**) 80 µg/mL) and evaluation of the respective hemoglobin content (**C**). ^a^ *p* < 0.001 vs. respective MPLE; ^b^ *p* < 0.001 vs. other MPFE; ^c^ *p* < 0.001 vs. other MPLE ^d^ *p* < 0.001 vs. CTR+; ^e^ *p* < 0.001 vs. CTR-.

Results of antiangiogenic activity, expressed as IC_50_ with respective C.L. at 95%, are depicted in Figure 8A. In panel B, it is possible to observe the representative stereomicroscopic images of the CAMs treated with vehicle (a), retinoic acid (10 μg/mL) as reference compound, and the highest concentrations of MPFE (c–e, for collection sites I, II and III, respectively) and MPLE (f–h, for collection sites I, II and III, respectively).

Observing the results from a qualitative point of view, more marked antiangiogenic activity of the MPFE II and MPLE II can be seen. However, although the IC_50_ values calculated for MPFE II and MPLE II are significantly lower than those for the extracts from sampling sites I and II, no statistically significant difference was found either between MPFE and MPLE, or between the different sampling sites (I, II and III).

These data were corroborated by the quantification of the hemoglobin content expressed as mg/g CAM (Figure 8C). Furthermore, in this case, no statistically significant difference was found either between MPFE and MPLE or between the different sampling sites (I, II and III), confirming data obtained through qualitative and semiquantitative analysis. However, it should be noted that all the extracts showed marked and statistically significant (*p* < 0.001) antiangiogenic activity with respect to the CTR-, reducing the hemoglobin content, on average, by 63% against the 75% of retinoic acid 10 µg/mL used as a reference standard (Figure 8C).

## 3. Discussion

In angiosperms, different kind of crystals have been found within organs and tissues of species from different families, in particular calcium oxalate, which is widely distributed in plants, and other less frequently present calcium salts, such as calcium tartrate in *Vitis* spp. or calcium malate in *Fraxinus excelsior* L. On the contrary, organic crystals only occur in specific plant families, e.g., inulin in *Asteraceae* and berberin in the *Berberidaceae* [27]. Flavonoid crystals are relatively infrequent and have been reported only in certain plant families or species, sometimes located in specific tissues. For example, the yellow tepals of some species of *Astrophytum* Lem. (*Cactaceae*) contain flavonol glycosides, together with flavonols in the form of spherical crystals [28,29]. Furthermore, needle-shaped crystals of hesperidin were detected in the peel cells of several *Rutaceae* species [30,31], and both hesperidin and diosmin crystals were found in leaves of *Crithmum maritimum* (*Apiaceae*) [32].

Diosmin is a flavonoid with multiple medicinal properties; it improves venous tone, increases lymphatic drainage, protects capillary bed microcirculation, reduces capillary permeability and inhibits inflammatory reactions [33]. Its anti-inflammatory activity against carrageenan-induced rat paw oedema at a dose of 600 mg/kg body weight was demonstrated by Farnsworth et al. [34], as early as 1976. Diosmin is abundant in the leaves of *Barosma betulina* (*Rutaceae*), known as Buchu [35], which is used in South African traditional medicine as a stimulant and diuretic and in Western phytotherapy for the treatment of urinary infections [36]. The chemical nature of the crystal aggregates, previously reported as hesperidin crystals [37,38], was clarified by Hegnauer [39], who stated that they are diosmin spherocrystals. In fact, crystals of these two flavonoids are very similar from the morphological point of view; therefore, chemical analyses are necessary to identify their chemical nature.

In the *Lamiaceae* family, diosmin spherocrystals are quite common [27,40]. They have been described in *Hyssopus officinalis* L. and have been reported to be abundant in several *Mentha* species, such as *M. arvensis* L., *M. pulegium* L. and *M.* × *rotundifolia* (L.) Huds. [41]. In the case of *M.* × *rotundifolia*, Bonzani [42] previously verified, from the chemical point of view, that these crystals are flavonoids of the line metabolism of diosmin. Nevertheless, in situ detection and identification of flavonoid crystals has only been achieved in a few cases [30,31,32]. In *M. arvensis*, *M. pulegium* and *M.* × *rotundifolia*, microscopic analysis made it possible to identify these crystals in the mesophyll, cortex and vascular tissues up to the epidermis and, in some cases, also within the non-glandular trichomes and into capitate glandular trichomes [41]. In addition, the authors described a novel type of capitate glandular trichome for the genus *Mentha*, i.e., with a unicellular and stipitate head, showing a lipophilic phase, an aqueous phase and diosmin crystals.

In our study, we also detected diosmin spherocrystals within the secretory cells of the peltate glandular trichomes of *M. pulegium* leaves. This represents a novel finding, as it is the first time that diosmin crystals have been detected within the peltate glandular trichomes of *Lamiaceae*.

Some studies have reported that salinity may cause substantial changes in volatile compounds, total phenolic content and antioxidant activities in *Lamiaceae* belonging to the *Salvia* genus [43,44]. Similarly, NaCl treatments increased the levels of total phenolics and flavonoids in different *Thymus* species [45,46]. Moreover, a recent study showed that under salinity stress, increased amounts of diosmin were found in the shoots of hyssop plant [47]. Increased synthesis and accumulation of polyphenols under salinity stress could be related to the role of these compounds in detoxification and plant protection against produced ROS. Our study on a wild population of *M. pulegium* growing in completely different environments confirms previously data obtained in other *Lamiaceae* species under NaCl treatments, namely that salinity increases the expression of the secondary metabolite diosmin. By examining the diosmin content in leaf extract + flower extract of the samples (MPI, MPII and MPIII from the three different locations), the highest diosmin content was found in sample I (94.07 mg/100 g DE), followed by samples II (76.22 mg/100 g DE) and III (70.21 mg/100 g DE). Our finding of a greater quantity of diosmin in MPI agrees with the characteristics of the plant growth site, i.e., a semiclosed Mediterranean coastal lagoon (Stagnone di Marsala, Trapani), with soils periodically flooded by oligotrophic, mesotrophic, eutrophic or sometimes brackish waters [48].

Today, it is well known that the expression of secondary metabolites increases in response to environmental stress (salinity, temperature, altitude, type of soil and amount of rainfall) as part of the plant defense mechanism with species-specific effects [49,50,51].

Phenolic acids and flavonoids also play a pivotal role in photoprotection. Indeed, UV radiation unbalances the plant redox status, and the plant is consequently forced to overexpress some secondary metabolites more than others to counteract the strong oxidative stress [50,52]. A recent study investigated the impact of growth sites on the phenolic contents and antioxidant activities of three Algerian species of *Mentha* [23]. Analyzing different *M. pulegium* ecotypes, the authors observed that plant growth in the sampling site characterized by mild summer and hot winter, situated at the lowest altitude, and with medium-type soil with a balanced texture (moderate humidity and non-saline) had the highest content of polyphenols and, consequently, showed the best antioxidant activity. This is in accordance with our data, which show the collection site II as the most promising from the biological point of view. On the contrary, the fluctuating phytochemical and biological behavior of the MPFE and MPLE from sampling site I can be explained thanks to a previous study, which showed how polyphenols increase following a single stress, such as salinity, but decrease when the plant is subjected to combined stress [53]. Sampling site I, in addition to being characterized by high salinity, is characterized by significantly lower rainfall and higher temperatures than the other two sampling sites (II and III). Moreover, this is the first study which demonstrates that salinity induces the expression of sugar-derived C6 acids (2-galloylgalactaric acid) in *M. pulegium*, as previously observed for other plants such as *Oryza sativa* L. [49].

Previous studies indicated the presence of several flavonoids in the genus, e.g., diosmin in *M. spicata*, *M. pulegium* and *M. rotundifolia* [54,55], whereas hesperidin was reported in *M. longifolia* [56], *M. piperita* [54] and *M. microphylla* [57]. An increase in rosmarinic acid was also observed in other plants, such as *Melissa officinalis* and *Dracocephalum kotschyi* Boiss. under heat and saline stress, respectively [58,59]. Generally, the biosynthesis of caffeic acid and its derivatives is enhanced under abiotic stress conditions, such as drought, heavy metal, salinity, high/low temperature and ultraviolet radiation [60].

Several studies showed that higher amounts of phenolic compounds and flavonoids are generally detected in plants sampled at higher altitudes [61]. 

The results of our study, in which, for the first time, both leaf and flower extracts of *M. pulegium* plants collected in three different sampling sites were analyzed, indicate that this phenomenon involves mainly flavonoids. These compounds were increased in sampling site III with respect to sampling site II in leaf extracts and even more in flower extracts, reflecting a completely different phytochemical profile compared to the other two sampling sites (I and II), and clearly unbalanced in favor of flavonoids. This is in accordance with previous results, which showed that chalcone synthase, the starting enzyme involved in the flavonoid biosynthesis pathway, is transcriptionally up-regulated by UV light [62,63]. Similarly, more recently studies, highlighted a substantial increase in dihydroxy B-ring-substituted flavonoids and flavonoids with UVB light-absorbing properties, such as kaempferol 7-*O*-rutinoside, in plants exposed to surplus solar radiation [61,64]. This could explain why the plants collected in sampling site III show a jaceidin isomer A chemotype and express mostly kaempferol derivatives, with respect to the others. Furthermore, according to previous data, the biological activity of the extracts from sampling site III well correlates with the expression of a specific metabolite, a riboflavin derivative, well-known for its strong antioxidant properties [65].

Temperature is another key parameter that has a significant influence on the biosynthesis of secondary metabolites. Our results demonstrate that low temperatures shift the metabolic pathways in favor of flavonoids, whereas, on the contrary, high temperatures favor the overexpression of phenolic acids [60]. However, as highlighted previously by Bautista et al. [61], the amount and type of phenolic acids and flavonoids can vary significantly, not only as a function of the pedoclimatic growth conditions but also based on the particularly complicated taxonomy of this genus. Mint includes approximately 42 species and 15 hybrids, with hundreds of subspecies and cultivars [1]. For example, within a single species, genetic factors influence considerablythe rosmarinic acid expression, sometimes losing the primacy of the most abundant compound according to the observations found in the present study for collection sites I and III [66]. This is of particular importance for *M. pulegium* because, among the most abundant secondary metabolites identified in the extracts under investigation, rosmarinic acid and its derivatives, lithospermic, yunnaneic, melitric and salvianolic acids, are the most powerful and investigated antioxidant and anti-inflammatory compounds, recognized for their outstanding antiangiogenic, antitumor and antimicrobial properties [67,68]. Rosmarinic acid is generally the most abundant compound in *Mentha* spp., but several chemotypes can be found, according to our results, depending on different abiotic or biotic selection agents [69].

The biological activity of the identified chemotype I could be attributable, other than to rosmarinic acid, to the presence of the most abundant compound kaempferide, for which interesting cytotoxic and anticancer properties, as well as chemosensitizing activities, have been previously described [70,71]. Finally, according to our results, which showed interesting antioxidant, anti-inflammatory and antiangiogenic properties for MPLE III and MPFE III, it was previously demonstrated that Jaceidin, the most abundant compound in these extracts, exhibits a strong cytotoxic effect in vitro on liver cancer and breast cancer cell lines. Therefore, it showed in vivo antitumor activity against Ehrlich’s ascites carcinoma, reducing the levels of vascular endothelial growth factor (VEGF) and consequently inhibiting VEGF-mediated angiogenesis, in addition to ameliorating the oxidative stress parameters [72].

Therefore, the selection of the most appropriate chemotype is a critical factor in conferring a specific phytotherapeutic application to the plant complex.

Although the most abundant compounds identified in the extracts from the three sampling sites have shown interesting antioxidant, anti-inflammatory and antiangiogenic properties, it is not possible to exclude other possible causes of these activities. Accordingly, the quantification of the most abundant constituents within the extracts, their isolation, and comparison of the biological activities found for the plant complexes with those of the most abundant compounds tested at the same concentrations present within the extracts, will be evaluated in a subsequent study. This will help to verify a possible synergistic effect of the main constituents with other minor compounds and to investigate and clarify a possible mechanism of action.

## 4. Materials and Methods

### 4.1. Plant Collection

Three plants of *M. pulegium* were harvested in June–July 2019 in three different locations in Sicily (Italy): Isola Lunga, Trapani; Castronovo di Sicilia, Palermo; and Castellana Sicula, Palermo (Table 4). One of the authors (F.M.R.) carried out the taxonomic identification. Voucher specimens (see Table 4 for specifications) were deposited in PAL-Gr.

### 4.2. Chemicals

Reagents, as well as American Chemical Society (ACS) and LC-MS-grade solvents, unless otherwise specified, were purchased from Merck (Darmstadt, Germany).

### 4.3. Micromorphological Analyses

Micromorphological features of nine mature *M. pulegium* flowers and leaves (MPF and MPL, respectively) collected in the three Sicilian locations were analyzed by light microscopy (LM) and scanning electron microscopy (SEM).

#### 4.3.1. Light Microscopy

Epidermal peels and handmade transversal sections of each sample were obtained using a razor blade and dissecting forceps. Samples were then mounted in distillate water and observed under a Leica D.M. 2000 microscope equipped with a digital camera (DFC 320, Leica Microsystems, Wetzlar, Germany). Samples of MPF and MPL were also formalin-fixed overnight at room temperature (RT), then processed with an automated Leica ESP 6025 processor and paraffin-embedded. Subsequently, the paraffin blocks were sectioned with a rotative microtome (Leica RM2265), obtaining sections with a thickness of 3–4 μm. Semithin sections were stained with Toluidine Blue O (TBO) as metachromatic staining for both polyphenols and polysaccharides detection [73], and with hematoxylin–eosin for general staining. Polarized light was used to detect crystals and starch grains [74].

#### 4.3.2. Scanning Electron Microscopy

Samples of MPL (1.5–2.0 cm^2^) and whole MPF were fixed in 70% ethanol-FineFix working solution (Milestone s.r.l., Bergamo, Italy) overnight at 4 °C and dehydrated through an ethanol series according to Chieco et al. [75]. Samples were then critical-point-dried in CO_2_ (CPD, K850 2 M Strumenti s.r.L., Rome, Italy). After critical-point drying, MPL were used whole or in handmade sections prepared using a razor blade. All samples were mounted on aluminum stubs, on glued carbon tabs, and coated with 10 nm gold. Subsequently, they were analyzed at a 20 kV accelerating voltage with a Vega3 Tescan LMU SEM-EDS Apollo XSD (Tescan USA Inc., Cranberry Twp, PA, USA).

### 4.4. Preparation of Hydroalcoholic Extracts

MPF (*n* = 3) and MPL (*n* = 3) of each plant (I, II and III) were manually separated and processed according to Smeriglio et al. [31] to obtain three independent hydroalcoholic extracts for each plant material (*n* = 9 leaf extracts and *n* = 9 flower extract for each sampling site). Briefly, fresh MPF and MPL samples were powdered by a blade analytical mill (A11, IKA^®^-Werke GmbH & Co. KG, Staufen, Germany) with liquid nitrogen to block the enzymatic activity and preserve the native phytochemical features. One hundred milliliters of a hydroalcoholic mixture (ethanol:water, 80:20 *v*/*v*) were added to each powdered flower and leaf sample (10 g), vortex-mixed for 3 min and sonicated in an ice-cold bath for 5 min using a 3 mm titanium probe set to 200 W and 30% amplitude (Vibra Cell™ Sonics Materials, inc., Danbury, Connecticut, USA). Extracts were centrifugated at 3000× *g* 15 min at 4 °C, and the supernatants, filtered on Whatman filter paper no. 1, were recovered in a round-bottom flask. Extraction was repeated two more times, and the supernatants were pooled and dried by rotary evaporator (Büchi R-205, Cornaredo, Italy). Dry extracts (DEs) were stored in a vacuum glass desiccator for 48 h with anhydrous sodium sulfate. After extraction and yield calculation (MPFE 18.11–22.23% and MPLE 15.47–20.31%), DEs were stored at −20 °C until subsequent analyses.

### 4.5. Phytochemical Screening

#### 4.5.1. Total Phenolics

Total phenolics were quantified according to Smeriglio et al. [31]. Briefly, 50 µL of MPLE and MPFE (0.25–2.0 mg/mL) were added to 450 µL of deionized water and Folin–Ciocalteu reagent (500 µL). After 3 min incubation, 10% sodium carbonate (500 µL) was added, incubating the samples in the dark at RT for 60 min, vortex-mixing every 10 min. Absorbance was read at 785 nm with an UV–Vis spectrophotometer (Model UV-1601, Shimadzu, Kyoto, Japan) against a blank consisting of ethanol:water, 80:20 *v*/*v*. Gallic acid was used as a reference compound (0.075–0.6 mg/mL), and results were expressed as g gallic acid equivalents (GAE)/100 g dry extract (DE).

#### 4.5.2. Total Flavonoids

Total flavonoids were quantified according to Smeriglio et al. [76]. Briefly, 0.2 mL of MPLE and MPFE (0.625–5.0 mg/mL) was mixed 1:1 (*v*:*v*) with 2 mg/mL AlCl_3_ and 1.2 mL of 50 mg/mL sodium acetate, and incubated for 2.5 h at RT. The absorbance was recorded at 440 nm using the same instrument and blank reported in Section 4.5.1. Rutin was used as a reference compound (0.125–1.0 mg/mL), and results were expressed as g rutin equivalents/100 g DE.

#### 4.5.3. Flavanols

Quantification of flavanols was carried out according to Monforte et al. [77]. Briefly, 2.0 mL of MPLE and MPFE (300 µg/mL) diluted in 0.5 M H_2_SO_4_ were loaded onto a conditioned Sep-Pak C18 cartridge (Waters, Milan, Italy), washed with 2.0 mL of H_2_SO_4_ (5.0 mM) and eluted with 5.0 mL of methanol. One milliliter of each eluate was added to 6.0 mL of 4% vanillin methanol solution and incubated in a water bath at 30 °C for 10 min. After cooling, 3 mL of HCl were added, and after 15 min incubation, the absorbance was recorded at 500 nm using the same instrument and blank reported in Section 4.5.1. Catechin was used as a reference compound (0.125–0.50 mg/mL). Results were expressed as g catechin equivalents (CE)/100 g DE.

### 4.6. Phytochemical Characterization by LC-DAD-ESI-MS Analysis

The phytochemical characterization of MPLE and MPFE was carried out by RP-LC-DAD-ESI-MS analysis according to Danna et al. [78]. Chromatographic analysis was carried out using a Luna Omega PS C18 column (150 mm × 2.1 mm, 5 µm; Phenomenex, Torrance, CA, USA) at 25 °C by with 0.1% formic acid (Solvent A) and methanol (Solvent B) as mobile phase according to the following elution program: 0–3 min, 0% B; 3–9 min, 3% B; 9–24 min, 12% B; 24–30 min, 20% B; 30–33 min, 20% B; 33–43 min, 30% B; 43–63 min, 50% B; 63–66 min, 50% B; 66–76 min, 60% B; 76–81 min, 60% B; 81–86 min, 0% B and equilibrated 4 min. Five microliters of each extract was injected, and the UV–Vis spectra of analytes were recorded in the range of 190 to 600 nm. Chromatograms were acquired at different wavelengths (260, 280, 292, 330, 370 and 520 nm) to identify all polyphenol classes. The experimental parameters of the ion trap (model 6320, Agilent Technologies, Santa Clara, CA, USA) were set as follows: negative (ESI-) ionization mode, 3.5 kV capillary voltage, 40 psi nebulizer (N_2_) pressure, 350 °C drying gas temperature, 9 L/min drying gas flow and 40 V skimmer voltage. Acquisition was carried out in full-scan mode (90–1000 m/z). Data were acquired by Agilent ChemStation software version B.01.03 and Agilent trap control software version 6.2. Identification was carried out by comparing the retention times and UV–Vis and MS spectra of each analyte with those of commercially available standards (when available, see Table 3), literature data and UV–Vis and mass spectra databases. The LC-DAD chromatogram acquired at 280 nm was used for quantification, expressing the results as mean area percentage (%) ± standard deviation of nine independent analyses in triplicate (*n* = 3) with respect to the total area of identified and unidentified (unknown) compounds. Diosmin quantification was carried out using an external calibration curve (1–50 µg/mL, R^2^ = 0.9997) built with a HPLC-grade (purity ≥ 99%) reference standard (Extrasynthase, Genay, France).

### 4.7. In Vitro Cell-Free Assays for Determination of Antioxidant and Anti-Inflammatory Properties

#### 4.7.1. DPPH Assay

The DPPH assay was carried out according to Smeriglio et al. [79] with some modifications. Briefly, 3.75 μL of MPLE and MPLE (6.0–400 µg/mL) was added to 150 µL fresh DPPH methanol solution (70 mg/L), mixed and incubated in the dark for 20 min. The absorbance was recorded at 517 nm against the blank reported in Section 4.5.1 using a UV–Vis plate reader (Multiskan GO; Thermo Scientific, Waltham, MA, USA). Trolox was used as reference standard (0.63–5.0 µg/mL). The results, which represent the average of nine independent experiments in triplicate (*n* = 3), were expressed as the inhibition percentage (%), calculating the IC_50_ with the respective confident limits (C.L.) at 95% by Litchfield and Wilcoxon’s test using PHARM/PCS software version 4 (MCS Consulting, Wynnewood, PA, USA).

#### 4.7.2. FRAP Assay

The ferric-reducing antioxidant power was evaluated according to Occhiuto et al. [80] with some modifications. Briefly, 10 microliters of MPLE and MPFE (2.5–100 μg/mL) was added to 200 µL of fresh, prewarmed (37 °C) working FRAP reagent consisting of 300 mM buffer acetate (pH 3.6), 10 mM 2,4,6-Tris (2-pyridyl)-s-triazine (TPTZ)-40 mM HCl and 20 mM FeCl_3_, and incubated for 4 min at RT in the dark. The absorbance was recorded at 593 nm using the same instrument and blank reported in Section 4.7.1. Trolox was used as reference compound (2.50–20.0 μg/mL). Results were expressed as reported in Section 4.7.1.

#### 4.7.3. TEAC Assay

The Trolox equivalent antioxidant capacity was evaluated according to Bazzicalupo et al. [81] with some modifications. The colored and stable cationic radical was generated in 12 h at RT and in the dark by oxidation of the diammonium salt of 2,2′-azino-bis (3-ethylbenzothiazolin-6-sulphonic acid (ABTS, 1.7 mM) by means of a solution of 4.3 mM potassium persulfate (K_2_S_2_O_8_). Then, the radical solution was diluted to obtain an absorbance of 0.7 ± 0.02 at 734 nm and used within 4 h. Ten microliters of MPLE and MPFE (2.5–100 μg/mL) was added to the reagent (200 µL) and incubated at RT for 6 min. The decrease in absorbance was recorded at 734 nm by using the same instrument and blank reported in Section 4.7.1. Trolox was used as a reference compound (1.25–10.0 μg/mL). Results were expressed as reported in Section 4.7.1.

#### 4.7.4. ORAC Assay

The ORAC assay was carried out according to Bellocco et al. [82]. Briefly, 20 μL of MPLE and MPFE (0.08–5.0 μg/mL) was added to 120 μL of fresh 117 nM fluorescein and incubated for 15 min at 37 °C. Then, 60 microliters of 40 mM AAPH was added to trigger the reaction, which was recorded every 30 s for 90 min (λ_ex_ 485; λ_em_ 520) by a fluorescence plate reader (FLUOstar Omega, BMG LABTECH, Ortenberg, Germany). Trolox was used as a reference compound (0.25–2.5 μg/mL). Results were expressed as reported in Section 4.7.1.

#### 4.7.5. Bovine Serum Albumin (BSA) Denaturation Assay

The ability of MPLE and MPFE to inhibit heat-induced BSA denaturation was evaluated according to Smeriglio et al. [83]. Briefly, 100 μL of 0.4% fatty-acid-free BSA solution and 20 μL of PBS (pH 5.3) were added to a 96-well plate. Then, 80 μL of MPLE and MPFE (30.0–500 μg/mL) was added, and the absorbance was immediately recorded at 595 nm (T_0_). Subsequently, samples were incubated for 30 min at 70 °C and, at the end, the absorbance was recorded again (T_30_) using the same instrument and blank reported in Section 4.7.1. Diclofenac sodium was used as a reference compound (3.0–24.0 μg/mL). Results were expressed as reported in Section 4.7.1.

#### 4.7.6. Protease Inhibition Assay

The protease inhibitory activity of MPLE and MPFE was evaluated according to Smeriglio et al. [83]. Briefly, 200 μL of MPLE and MPFE (20.0–160.0 μg/mL) were added to a reaction mixture consisting of 10 μg/mL trypsin (12 μL) and 25 mM Tris-HCl buffer (pH 7.5, 188 μL). Two-hundred microliters of 0.8% casein were added and the reaction mixture and incubated for 20 min at 37 °C in a water bath. Then, 400 μL of perchloric acid were added to stop the reaction. The cloudy suspension was centrifuged at 3500× *g* for 10 min, and the absorbance of the supernatant was recorded at 280 nm by an UV–Vis spectrophotometer (Shimadzu UV 1601, Kyoto, Japan) against the same blank reported in Section 4.7.1. Diclofenac sodium was used as reference compound (2.0–16.0 μg/mL). Results were expressed as reported in Section 4.7.1.

### 4.8. In Vitro Cell-Based Assays for Determination of Antioxidant and Anti-Inflammatory Properties

Antioxidant and anti-inflammatory activity were also evaluated on human primary cells (erythrocytes and peripheral blood mononuclear cell) collected and processed according to Smeriglio et al. [84].

#### 4.8.1. Heat-Induced Hemolysis Assay

The hemolysis assay was carried out according to Smeriglio et al. [84]. Briefly, 45 μL of packed erythrocytes were added to 45 μL of MPLE and MPFE (20–80 µg/mL), brought to a volume of 3 mL with 10 mM PBS (pH 7.4) and incubated under shaking (120 rpm) for 60 min at 37 °C by an Innova 4000 Benchtop Incubator Shaker (New Brunswick Scientific, Edison, NJ, USA). The mixture was centrifuged at 2000× *g* for 3 min at RT, and the absorbance of the supernatant was recorded at 540 nm by a reader plate (Multiskan GO; Thermo Scientific, MA, United States). Deionized water and 10 mM PBS instead of sample were used as positive (100% hemolysis) and negative control, respectively. Diclofenac sodium (50.0–200.0 µg/mL) was used as reference compound. Results were expressed as reported in Section 4.7.1.

#### 4.8.2. Scavenging Activity against Intracellular ROS

The radical-scavenging ability of MPLE and MPFE against intracellular ROS was evaluated according to Smeriglio et al. [84]. Packed erythrocytes were suspended in 10 mM PBS (pH 7.4) to obtain 1% erythrocyte suspension. Then 10 µL of MPLE and MPFE (2.5–20 µg/mL) were added to 1 mL of erythrocyte suspension, mixed and incubated for 2 h in the dark at 20 °C under shaking (120 rpm, see Section 4.8.1 for incubator shaker specifications). Subsequently, samples were centrifuged at 2400× *g* for 5 min, and the erythrocyte pellet was washed three times with 10 mM PBS (pH 7.4, 1:1, *v*/*v*) to remove any residue of extracellular antioxidant compounds. After cell pellet lysis by deionized water (0.1 mL), 1 mL of 2′,7′ -dichlorofluorescin diacetate (DCF-DA, D6883, Merck, Darmstadt, Germany) working solution (0.28 μg/mL) and 167 mM hydrogen peroxide solution (18.9 μL) were added, and ROS were detected recording the fluorescence intensity after 10 min (λ_ex_ 485; λ_em_ 535). Trolox (12.50–50.0 μg/mL) and PBS were used as positive and negative controls, respectively. Results were expressed as reported in Section 4.7.1.

#### 4.8.3. Evaluation of Anti-Inflammatory Activity on Peripheral Blood Mononuclear Cell

The anti-inflammatory activity of MPLE and MPFE was evaluated according to Smeriglio et al. [84]. Briefly, 150 μL of fresh PBMC suspension was seeded in a 96-well plate (Nunc^®^, Merck, Darmstadt, Germany). A pretreatment was carried out by adding 20 μL of MPLE (5.0–20.0 µg/mL) and MPFE (2.5–10.0 µg/mL) in each well and incubated for 1 h at 37 °C under 5% CO_2_. Inflammation was induced by 10 ng/mL LPS (30 μL) and samples were incubated for 16 h at 37 °C 5% CO_2_. Diclofenac sodium (12.5–50 μg/mL) and 10 mM PBS (pH 7.4) were used as positive (CTR+) and negative controls (CTR-), respectively. Moreover, a blank consisting of cell suspension with 0.1% DMSO solution instead of sample was run in each assay. At the end of incubation time, cell supernatants were harvested, and interleukin-6 (IL-6) and tumor necrosis factor α (TNF-α) release (pg/mL) were recorded by highly sensitive human ELISA kits (DRG Diagnostics GmbH, Marburg, Germany), according to the manufacturer’s recommendations.

### 4.9. Chick Chorioallantoic Membrane (CAM) Assay for Determination of Antiangiogenic Properties

Antiangiogenic effects were evaluated using the CAM assay according to Smeriglio et al. [85]. Briefly, fertilized eggs of *Gallus gallus* were incubated for 4 days in a humidified incubator at 37 °C. Then, a window (1 cm^2^) was carefully created on the apical side of the egg to visualize the CAM. Eggs with malformed or dead embryos were discarded. MPLE and MPFE solutions, properly diluted in albumen (20–80 and 10–40 μg/mL), were applied directly on the CAM surfaces. Retinoic acid (10 μg/mL) was used as CTR+. CAMs treated only with the sample vehicle were also included as negative controls (CTR-). After treatment, the eggs were incubated for 24 h. The number of blood vessel branch points was evaluated in a standardized area by a stereomicroscope (SMZ-171 Series, Motic). Pictures were acquired by a digital camera (Moticam^®^ 5 plus) and analyzed by the GNU Image Manipulation Program (GIMP version 2.10.2). Results were expressed as reported in Section 4.7.1.

To corroborate the previous data, the hemoglobin (Hb) content, which is a sensitive index of vascular density, was evaluated in the treated CAMs according to Certo et al. [86]. CAM tissues were homogenized for 1 min in KCl (1.15%, *w*/*v*) with an IKA ULTRA-TURRAX^®^ T45 (IKA^®^-Werke GmbH & Co. KG, Staufen, Germany). The homogenate (20 μL) was mixed with Drabkin’s reagent (5 mL) and incubated for 5 min in the dark at RT. The absorbance was recorded at 540 nm using the same instrument reported in Section 4.7.6. Results were expressed as mg of Hb/g of CAM, using an external standard calibration curve (10–180 mg/mL).

### 4.10. Statistical Analysis

Nine independent experiments in triplicate (*n* = 3) for in vitro cell-free and cell-based experiments, and nine independent experiments in quintuplicate (*n* = 5) for in vivo experiments were carried out. The statistical significance was evaluated by one-way analysis of variance (ANOVA) followed by Student–Newman–Keuls and Tukey’s test using SigmaPlot 12.0 software (Systat Software Inc., San Jose, CA, USA). *p* < 0.05 was considered statistically significant.

## 5. Conclusions

Micromorphological analyses highlighted the presence of spheroidal crystals of diosmin in the leaf epidermal cells and along the leaf veins, which were also visible inside the thin petals of the flowers. In addition, diosmin spherocrystals were detected for the first time within the secretory cells of the peltate glandular trichomes of *M. pulegium* leaves. Different environments influence the diosmin content in the plant tissues; in particular, salinity increases the expression of this secondary metabolite in the plants growing on clayey saline depressions near the sea. Accordingly, MPI is the richest in flavonoids, whereas MPII is the richest in total phenolics.

Although some differences in the phytochemical profile of hydroalcoholic extracts of leaves and flowers were found, the most abundant polyphenols identified by LC-DAD-ESI-MS analysis were kaempferide (26.40%) in MPI, rosmarinic acid (22.12%) in MPII and jaceidin isomer A (27.58%) in MPIII. MPII showed the strongest antioxidant and anti-inflammatory activity, followed by MPIII and MPI. MPII also showed the strongest antiangiogenic activity, followed by MPI and MPIII.

In conclusion, pedoclimatic features influence considerably the phytochemical profile and the biological activity of *M. pulegium*, and the use of the flowering tops of this plant species finds a rationale in enhancing the biological activity of the plant complex. Finally, the kaempferide/rosmarinic acid chemotype is the most interesting from the biological point of view.

## Figures and Tables

**Table 1:** Comparison between micromorphological and phytochemical analyses of the diosmin content of *M. pulegium* leaves and flowers of plants collected in three different Sicilian locations: Isola Lunga-Stagnone di Marsala (MPI), Castronovo di Sicilia (MPII) and Castellana Sicula (MPIII).

Sample	Micromorphological Analysis		LC-DAD Analysis *	
	Abundance of crystals		mg diosmin/100 g DE ^a^	
	Leaves	Flowers	Leaves	Flowers
MPI	+++	+++	66.60 ± 0.44 ^b^	27.47 ± 0.24 ^b^
MPII	++	++	65.76 ± 0.21	10.46 ± 0.12 ^c^
MPIII	++	+	65.60 ± 0.33	4.61 ± 0.08

^a^ DE, dry extract; ^b^ *p* < 0.05 vs. MPII and MPIII; ^c^ *p* < 0.001 vs. MPIII; * acquired at 280 nm.

**Table 2:** Phytochemical screening by colorimetric assays. Results, which are reported as the mean ± standard deviation of nine independent experiments in triplicate (*n* = 3), are expressed as g of gallic acid equivalents (GAE)/100 g of dry extract (DE), g of rutin equivalents (RE)/100 g DE and g of catechin equivalents (CE)/100 g DE for total phenolics, flavonoids and flavan-3-ols, respectively.

Sample	Total Phenolics (g GAE/100 g DE)	Flavonoids (g RE/100 g DE)	Flavan-3-ols (g CE/100 g DE)
MPLE I	3.39 ± 0.32 ^a^	22.83 ± 1.42 ^a,b^	8.48 ± 0.23 ^a,b^
MPLE II	7.87 ± 0.72 ^b^	6.13 ± 0.18 ^b^	2.33 ± 0.11 ^b^
MPLE III	3.96 ± 0.28	6.54 ± 0.11	3.50 ± 0.21
MPFE I	11.15 ± 0.42 ^a,b^	23.73 ± 1.35 ^a,b^	2.75 ± 0.17 ^b^
MPFE II	8.87 ± 0.80 ^b^	6.53 ± 0.14 ^b^	2.86 ± 0.15 ^b^
MPFE III	5.21 ± 0.20	27.62 ± 2.63	4.75 ± 0.22

^a^*p* < 0.001 vs. MPII; ^b^ *p* < 0.001 vs. MPIII.

**Table 3:** Phytochemical profile of *M. pulegium* leaf and flower extracts (MPLE and MPFE, respectively) elucidated by LC-DAD-ESI-MS analysis. Results are expressed as mean area percentage (%) based on LC-DAD data acquired at 280 nm ± standard deviation of nine independent analyses in triplicate (*n* = 3) with respect to the total area of identified and unidentified (unknown) compounds.

n.	Compound	λ_max_ nm	[M-H]^-^ m/z	MPLE (Area %)			MPFE (Area %)		
				I	II	III	I	II	III
1	Riboflavine 5’-(dihydrogen phosphate)	300	455	-	3.34 ± 0.16 ^b^	**19.23 ± 0.56**	-	-	**7.31 ± 0.33**
2	Epicatechin hydroxybenzoate	272	409	-	-	0.73 ± 0.03	1.10 ± 0.04 ^a^	0.29 ± 0.01 ^b^	0.26 ± 0.01
3	Gallocatechin-3-gallate ^§^	278	457	-	-	-	-	0.70 ± 0.03	-
4	Epigallocatechin-3-*O*-gallate ^§^	274	457	0.37 ± 0.01	-	-	-	-	-
5	Floridin	256,282	414	-	-	2.48 ± 0.08	-	-	-
6	Caffeoylhydroxycitric acid	236,326	369	-	-	-	-	-	3.68 ± 0.15
7	Ferulic acid hexoside	234,322	355	-	-	-	0.68 ± 0.02	-	-
8	Catechin hexoside	276,320	451	-	0.28 ± 0.01	-	-	0.13 ± 0.00	-
9	Coumaroylquinic acid	216,320	338	0.72 ± 0.02	-	-	-	-	-
10	Salvianolic acid J	276,344	537	-	-	0.42 ± 0.02	-	-	-
11	Epicatechin methylgallate	278	455	-	-	-	0.38 ± 0.01	-	-
12	Malonyl-daidzin	264,308	297	-	-	0.72 ± 0.01	-	-	**8.3 ± 0.27**
13	Luteolin malonyl-hexoside	263,348	533	0.88 ± 0.03	-	-	-	-	-
14	Apigenin apiosyl-hexoside	268,328	563	-	5.43 ± 0.17	-	-	3.37 ± 0.12	-
15	Unknown	300	567	-	-	2.98 ± 0.12	-	-	0.98 ± 0.03
16	Unknown	300	391	-	-	1.13 ± 0.03	-	-	1.35 ± 0.05
17	Epitheaflagallin 3-*O*-gallate ^§^	260,294	551	-	-	-	-	0.62 ± 0.03 ^b^	**5.35 ± 0.18**
18	Epigallocatechin ^§^	278	305	-	-	-	2.07 ± 0.08	-	-
19	Gallocatechin ^§^	278	305	**8.31 ± 0.15 ^a^**	1.99 ± 0.07 ^b^	0.64 ± 0.02	-	-	-
20	Eriodictyol-7-*O*-glucoside	296,326	449	-	-	-	-	0.99 ± 0.02	-
21	Medioresinol ^§^	278	387	-	-	0.15 ± 001	-	-	-
22	Dimethylepigallocatechin gallate	278	485	-	-	**3.52 ± 0.12**	-	-	1.91 ± 0.05
23	Eriodictyol ^§^	292,328	287	-	-	-	-	0.38 ± 0.01	-
24	Tuberonic acid hexoside	274,304	387	-	1.17 ± 0.04 ^b^	0.96 ± 0.02	0.35 ± 0.01 ^a^	1.05 ± 0.03 ^b^	1.68 ± 0.04
25	Methyl-galloylgalactarate	226,268	375	2.27 ± 0.05	-	-	-	-	-
26	Hydroxyluteolin-xyloside	-	433	-	-	-	-	-	0.20 ± 0.01
27	Trihydroxy-methoxy-MDF-glucuronide	274,314	519	-	0.42 ± 0.02 ^b^	1.08 ± 0.04	-	-	0.47 ± 0.02
28	Epigallocatechin digallate	278	609	-	-	-	0.63 ± 0.02 ^a^	0.38 ± 0.02	-
29	Unknown	-	374	-	-	-	-	-	0.30 ± 0.02
30	Limocitrin^§^	280,328,379	375	-	-	-	-	0.49 ± 0.03 ^b^	0.24 ± 0.01
31	Kaempferol 3-*O*-glucuronide ^§^	272,368	461	-	-	-	-	-	**5.28 ± 0.12**
32	Luteolin 7-*O*-rutinoside ^§^	272,344	593	2.89 ± 0.03 ^a^	2.37 ± 0.14 ^b^	**5.36 ± 0.25**	2.98 ± 0.17 ^a^	1.65 ±0.04 ^b^	0.27 ± 0.02
33	Kaempferol 3-*O*-rutinoside ^§^	266,323,364	593	-	-	-	-	-	0.55 ± 0.02
34	Unknown	250,294	559	-	-	-	-	0.25 ± 0.01	-
35	Salvianolic acid G	258,346	417	0.30 ± 0.01 ^a^	0.78 ± 0.04 ^b^	1.37 ± 0.02	-	2.40 ± 0.12 ^b^	1.05 ± 0.24
36	Unknown	256,326	639	-	0.34 ± 0.02	-	-	-	-
37	Salvianolic acid K	286,321	555	-	-	-	-	-	0.75 ± 0.02
38	Ellagic acid acetyl pentoside	256,326	475	-	0.31 ± 0.01 ^b^	0.59 ± 0.02	-	0.22 ± 0.01	-
39	5-Nonadecylresorcinol	275	375	-	-	-	-	-	0.25 ± 0.01
40	Quercetin acetyl hexoside	262,354	505	-	-	-	0.96 0.03	-	-
41	Todolactol A	236,288	375	-	-	-	-	-	**6.71 ± 0.22**
42	Limocitrol	280,327,390	375	-	2.0 ± 0.08 ^b^	2.54 ± 0.08	-	1.51 ±0.05	-
43	Caffeoyl-rosmarinic acid	284,328	537	-	-	-	-	0.15 ± 0.00	-
44	Valoneic acid dilactone	298	469	-	-	-	-	-	0.5 ± 0.02
45	Syringic acid hexose	240,318	359	0.83 ± 0.04	-	-	-	-	-
46	Unknown	282,344	491	-	0.23 ± 0.01	-	-	-	-
47	Quercetin 3-*O*-(6’’-*O*-malonyl-β-D-glucoside) ^§^	262,352	549	-	-	1.34 ± 0.03	-	-	0.85 ± 0.03
48	Unknown	300	442	-	-	-	-	0.09 ± 0.00	-
49	Salvianolic acid I	252,318,352	537	3.15 ± 0.05 ^a^	**8.41 ± 0.22**	-	**6.82 ± 0.22 ^a^**	**8.76 ± 0.32**	-
50	Dihydroquercetin 3-rhamnoside ^§^	254,368	449	-	-	0.37 ± 0.02	-	-	-
51	Apigenin 6,8-di-C-glucoside ^§^	244,266,334	593	0.72 ± 0.02 ^a^	-	1.35 ± 0.03	2.01 ± 0.12 ^a^	-	1.50 ± 0.05
52	Salvianolic acid D ^§^	254,347	417	0.64 ± 0.01 ^a^	3.41 ± 0.11 ^b^	0.82 ± 0.02	-	0.67 ± 0.02 ^b^	1.24 ± 0.04
53	Myricetin acetylhexoside	240,374	521	-	-	1.28 ± 0.04	-	-	-
54	Unknown	248,312,364	513	-	-	0.81 ± 0.04	-	-	-
55	Galloylgalactaric acid	209,275	361	**8.71 ± 0.11**	-	-	-	-	-
56	Daidzin ^§^	252,312	415	-	-	1.17 ± 0.02	-	-	0.92 ± 0.02
57	Unknown	240,310	559	-	-	0.45 ± 0.01	-	-	-
58	3,4-Dicaffeoylquinic acid ^§^	216,326	515	-	-	2.08 ± 0.13	**11.64 ± 0.67 ^a^**	-	2.06 ± 0.08
59	Salvianolic acid B ^§^	244,285,336	717	-	0.25 ± 0.01	-	5.50 ± 0.22 ^a^	**6.85 ± 0.28**	-
60	Isosalvianolic acid B	254,285,340	717	-	-	-	0.79 ± 0.03 ^a^	**6.41 ± 0.32**	-
61	Syringaresinol ^§^	240,271	417	-	-	-	-	-	1.22 ± 0.04
62	Diosmin ^§^	260,350	607	**3.61 ± 0.06 ^a^**	1.23 ± 0.03 ^b^	**3.33 ± 0.14**	0.54 ± 0.02 ^a^	0.11 ± 0.00 ^b^	0.15 ± 0.01
63	Unknown	246,266,312	424	-	-	-	-	0.20 ± 0.01	-
64	Salvianolic acid K	250,270,345	555	-	0.61 ± 0.03 ^b^	2.95 ± 0.11	-	0.25 ± 0.01	-
65	Unknown	300	553	-	-	0.12 ± 0.00	-	-	-
66	Rosmarinic acid ^§^	292,332	359	0.52 ± 0.02 ^a^	**19.16 ± 0.48 ^b^**	0.26 ± 0.01	**24.31 ± 0.84 ^a^**	**25.52 ± 0.76**	-
67	Malonylglycitin	256,320	531	-	-	0.46 ± 0.02	-	-	-
68	Pentacosenylresorcinol	300	457	-	-	-	-	-	0.63 ± 0.02
69	Salvianolic acid E ^§^	286,314,350	717	-	**6.08 ± 0.12**	-	1.94 ± 0.06 ^a^	1.74 ± 0.04	-
70	Unknown	246,266,312	506	-	-	1.51 ± 0.04	-	0.05 ± 0.00	-
71	Lithospermic acid ^§^	238,336	537	-	-	-	1.59 ± 0.04 ^a^	0.37 ± 0.02	-
72	Unknown	286,323	519	-	1.01 ± 0.02	-	-	-	-
73	Salvianolic acid L	286,314,340	717	-	-	-	2.33 ± 0.08	-	-
74	Glucoerucin ^§^	217,236	419	-	-	0.6 ± 0.03	-	0.31 ± 0.01	-
75	3,5-Dicaffeoylquinic acid ^§^	216,322	515	-	0.22 ± 0.01	-	-	-	0.74 ± 0.03
76	Quercetin hydroxybenzoylhexoside	257,358	583	2.37 ± 0.04	-	-	-	-	-
77	Unknown	266,300,334	531	0.26 ± 0.01	-	-	-	-	-
78	Isorhamnetin sulfate	253,370	395	-	-	0.83 ± 0.02	-	-	1.23 ± 0.04
79	Apigenin 8-*C*-α-L-arabinoside 6-*C*-β-D-glucoside ^§^	268,333	563	-	-	0.27 ± 0.01	-	-	-
80	Rosmanol ^§^	262,288	345	-	-	-	-	5.51 ± 0.22	-
81	Syringetin ^§^	284,370	345	-	0.22 ± 0.00	-	0.13 ± 0.01	-	-
82	Salvianolic acid C ^§^	264,342	491	-	0.22 ± 0.01 ^b^	1.89 ± 0.04	**6.04 ± 0.22 ^a^**	0.19 ± 0.01 ^b^	0.88 ± 0.03
83	Isorhamnetin 3-glucuronide ^§^	260,370	491	-	**8.04 ± 0.32**	-	-	-	0.45 ± 0.02
84	Unknown	222,290,362	581	0.28 ± 0.01	-	-	-	-	-
85	Luteolin ^§^	254,266,346	285	**3.65 ± 0.15**	-	-	-	-	-
86	Mangiferin gallate	260,362	573	-	0.11 ± 0.00	-	-	-	-
87	Peonidin ^§^	282,526	300	-	-	-	0.78 ± 0.04 ^a^	0.58 ± 0.04	0.65 ± 0.03
88	Patuletin hexoside	262,354	493	-	0.42 ± 0.02	-	-	-	-
89	Jaceosidin ^§^	214,282,342	329	-	4.44 ± 0.12 ^b^	1.42 ± 0.05	1.35 ± 0.03 ^a^	3.50 ± 0.14 ^b^	2.6 ± 0.01
90	Unknown	216,286,402	328	-	-	-	-	0.09 ± 0.00	-
91	Unknown	288,312,398	598	-	-	-	1.01 ± 0.02	-	-
92	Diosmetin 7-glucuronide ^§^	230,266,346	475	-	-	-	-	0.05 ± 0.00	-
93	Salvigenin ^§^	284,334	327	-	0.37 ± 0.01 ^b^	0.96 ± 0.03	0.76 ± 0.03	-	1.15 ± 0.02
94	Jaceidin Isomer A	282,352	359	**4.84 ± 0.18 ^a^**	**14.47 ± 0.58 ^b^**	**23.63 ± 1.05**	**15.73 ± 0.83 ^a^**	**13.45 ± 0.48 ^b^**	**30.79 ± 1.58**
95	Kaempferide ^§^	254,266,344	299	**52.81 ± 0.64**	-	-	-	-	-
96	Jaceosidin Isomer I	252,268,346	329	-	1.00 ± 0.04 ^b^	0.27 ± 0.01	0.73 ± 0.03 ^a^	0.21 ± 0.01 ^b^	1.51 ± 0.05
97	Quercetin sulfate	246,354	381	-	-	-	-	1.77 ± 0.05	-
98	Jaceidin isomer II	270,354	359	-	1.74 ± 0.03 ^b^	0.76 ± 0.03	0.64 ± 0.02 ^a^	-	1.40 ± 0.04
99	Phloretin xylosyl-hexoside	230,288	567	-	-	0.49 ± 0.02	-	-	0.36 ± 0.01
100	6-Methoxyapigenin ^§^	260,330	299	-	0.32 ± 0.01	-	-	-	-
101	Unknown	254,264,348	344	-	-	0.65 ± 0.03	-	-	-
102	Apigenina-7-glucoside ^§^	268,330	431	0.72 ± 0.03	-	-	-	-	-
103	Methyl digalloyl-β-D-hexopyranoside	268,310,360	497	0.59 ± 0.01	-	-	-	-	-
104	Dihydroxyphenyl galloyl-β-D-hexopyranoside	252,290,342	439	0.56 ± 0.02 ^a^	**7.14 ± 0.21 ^b^**	0.45 ± 0.02	**6.21 ± 0.02 ^a^**	**8.27 ± 0.14 ^b^**	0.96 ± 0.02
105	5-Galloylquinic acid ^§^	-	343	-	0.28 ± 0.01	-	-	-	-
106	Trachelogenin ^§^	-	387	-	0.07 ± 0.00	-	-	-	-
107	Unknown	-	381	-	-	-	-	0.06 ± 0.00	-
108	Hexopyranuronosyl-xylose	266,334	325	-	0.13 ± 0.00	-	-	0.09 ± 0.00 ^b^	0.69 ± 0.03
109	Quercetin 4’-glucuronide ^§^	258,368	477	-	-	0.64 ± 0.02	-	-	-
110	Unknown	266,332	488	-	-	-	-	0.06 ± 0.00	-
111	Epigallocatechin-caffeate	274,344	467	-	1.56 ± 0.03 ^b^	**4.0 ± 0.21**	-	-	-
112	Unknown	276,340	675	-	0.43 ± 0.01 ^b^	0.94 ± 0.02	-	0.26 ± 0.01 ^b^	0.63 ± 0.02

^§^ Absolute identification by commercially available reference standards purchased from Merck (Darmstadt, Germany), Extrasynthase (Geney, France) and MedChemExpress (Sollentuna, Sweden); - not detected; ^a^ *p* < 0.001 vs. MPII and MPIII; ^b^ *p* < 0.001 vs. MPIII.

**Table 4:** Data on collection sites of *M. pulegium* L. wild populations in the Sicily region (*n* = 3 for each collection site).

Sample	Collection Site	Altitude (m a.s.l) ^a^	GPS Coordinates	T ^b^ (°C)	P ^c^ (mm/year)	Soil	Voucher
MPI	Isola Lunga- Stagnone di Marsala (Trapani)	2.00	37°54′06″ N, 12°27′07″ E	18.5	520	Clayey-saline depressions	R&Sp ^d^ 05/19
MPII	Castronovo di Sicilia (Palermo)	338	37°40′09″ N, 13°38′54″ E	16.5	700	Clayey-moist	R&Sc ^e^ 06/19
MPIII	Castellana Sicula (Palermo)	860	37°45′42″ N, 13°59′73″ E	16.0	800	Clayey-moist	R&Sc ^e^ 07/19

^a^ m a.s.l., meters above sea level; ^b^ T, average annual temperature; ^c^ P, precipitation; ^d^ R&Sp, Raimondo and Spadaro; ^e^ R&Sc, Raimondo and Schimmenti.

## Data Availability

Not applicable.

## Supplementary Materials

The following supporting information can be downloaded at: https://www.mdpi.com/article/10.3390/plants12010024/s1, Figure S1: Representative LC-MS chromatogram of *M. pulegium* leaf extract I (MPLE I). Peak numbers refer to compounds listed in Table 3; Figure S2: Representative LC-MS chromatogram of *M. pulegium* leaf extract II (MPLE II). Peak numbers refer to compounds listed in Table 3; Figure S3: Representative LC-MS chromatogram of *M. pulegium* leaf extract III (MPLE III). Peak numbers refer to compounds listed in Table 3; Figure S4: Representative LC-MS chromatogram of *M. pulegium* flower extract I (MPFE I). Peak numbers refer to compounds listed in Table 3; Figure S5: Representative LC-MS chromatogram of *M. pulegium* flower extract II (MPFE II). Peak numbers refer to compounds listed in Table 3; Figure S6: Representative LC-MS chromatogram of M. pulegium flower extract III (MPFE III). Peak numbers refer to compounds listed in Table 3.
