# Supplementary material for: Pedoclimatic Conditions Influence the Morphological, Phytochemical and Biological Features of Mentha pulegium L
Source: Plants (Basel). 2022 Dec 21;12(1):24. doi: 10.3390/plants12010024 (PMC9824027; doi:10.3390/plants12010024)
Supplement: Supplementary file 1 [file plants-12-00024-s001.zip › plants-2068352-supplementary.pdf]

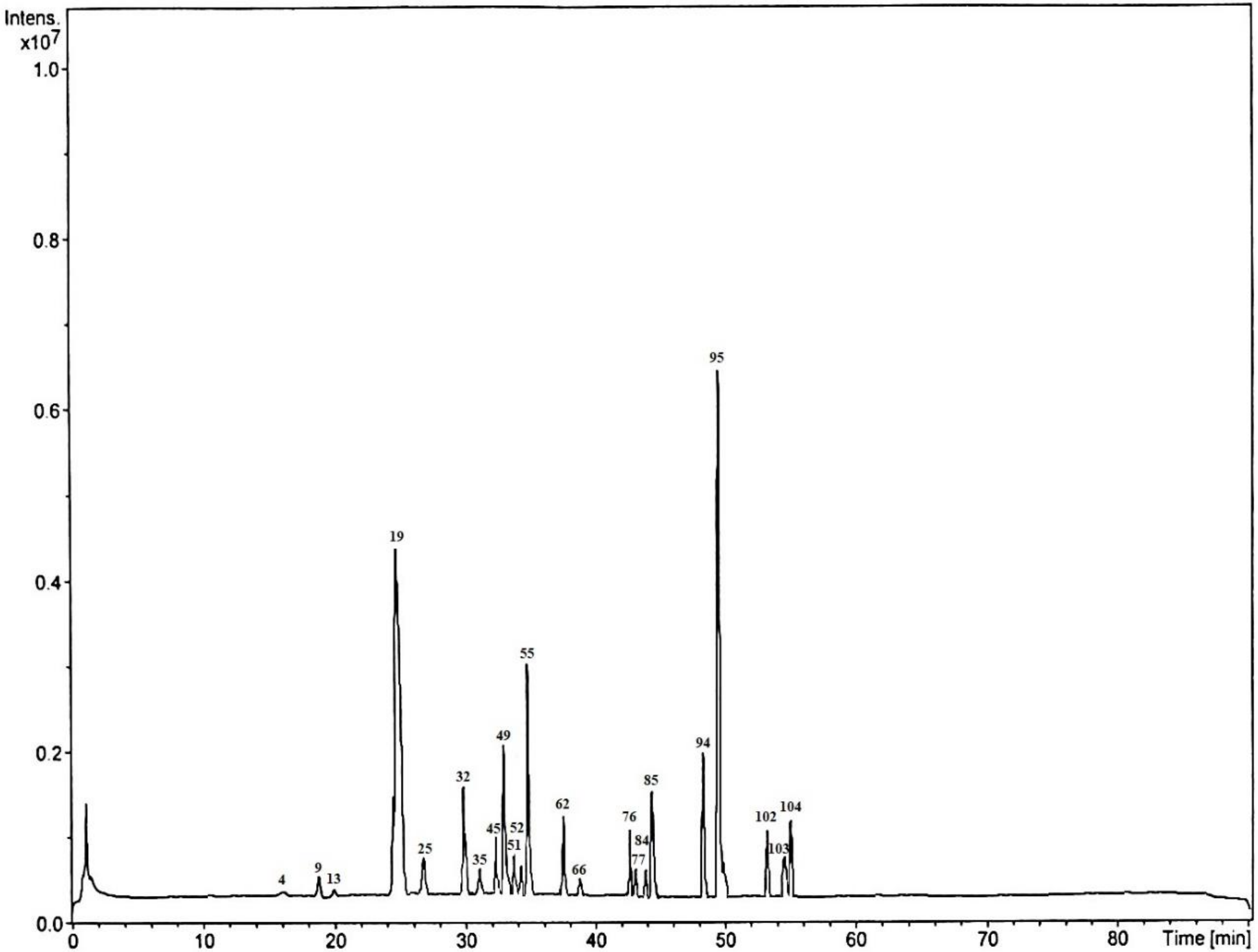

Figure S1. Representative LC-MS chromatogram of *M. pulegium* leaf extract I (MPLE I). Peak numbers refer to compounds listed in Table 3.

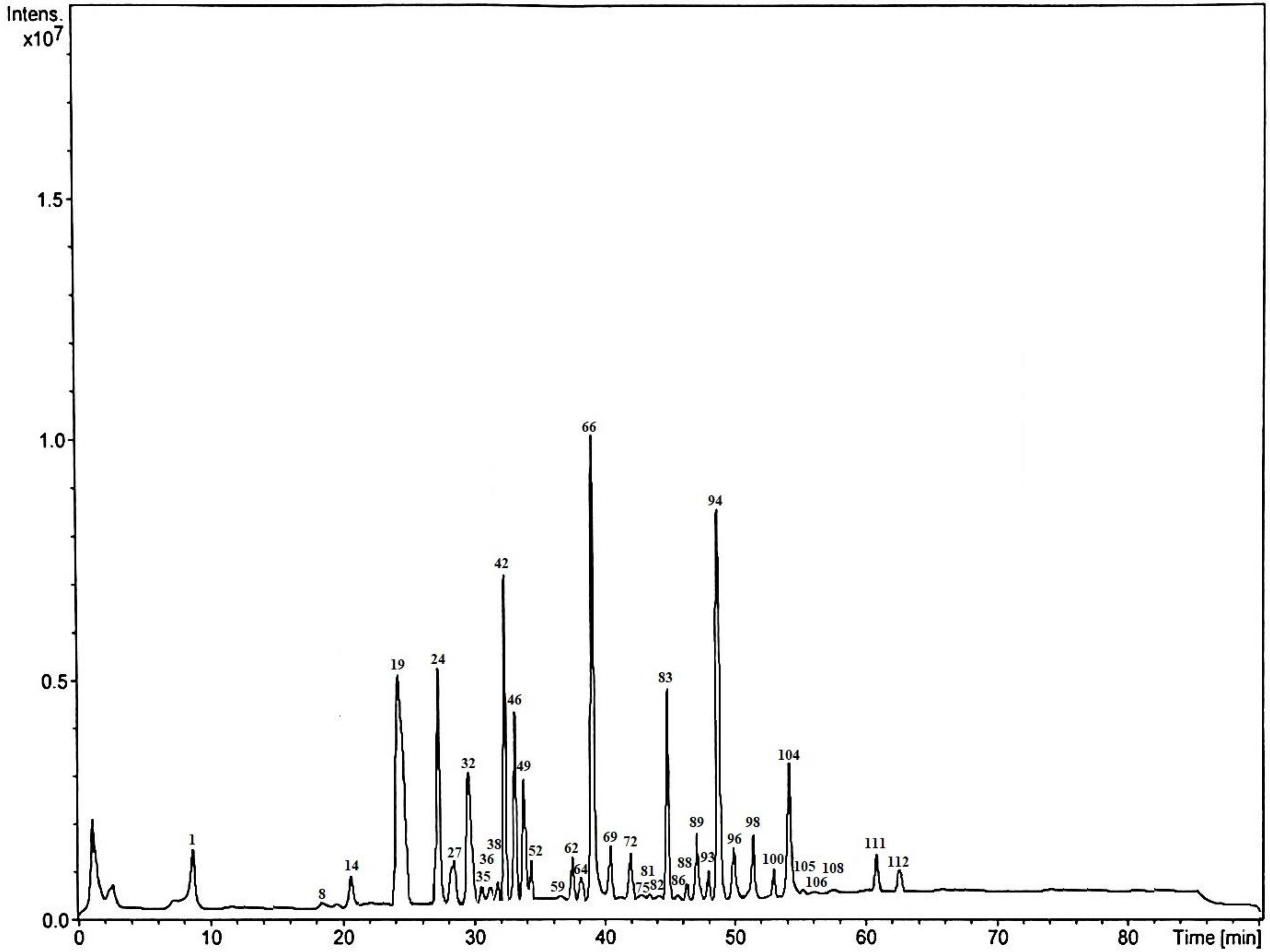

Figure S2. Representative LC-MS chromatogram of *M. pulegium* leaf extract II (MPLE II). Peak numbers refer to compounds listed in Table 3.

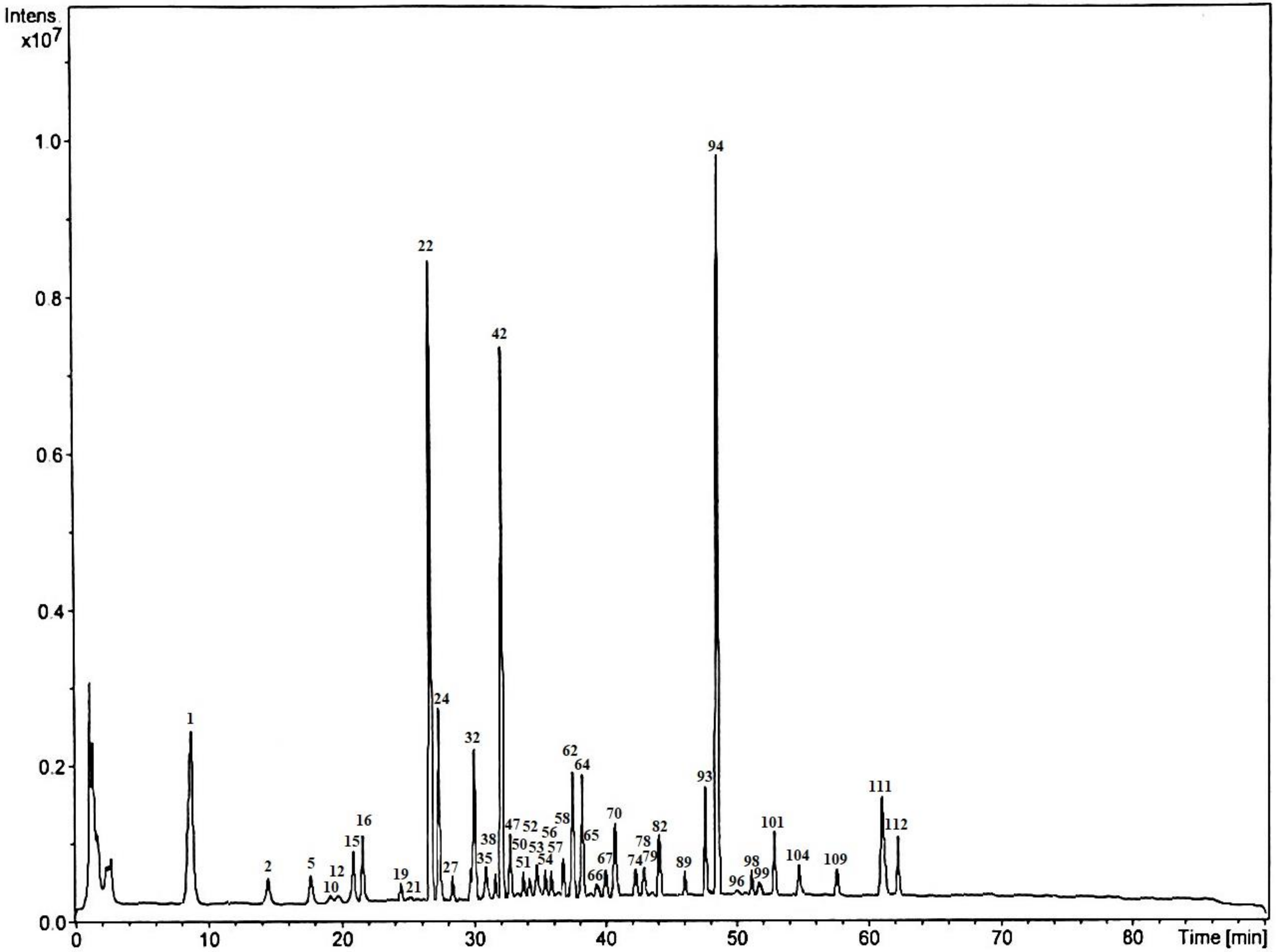

Figure S3. Representative LC-MS chromatogram of *M. pulegium* leaf extract III (MPLE III). Peak numbers refer to compounds listed in Table 3.

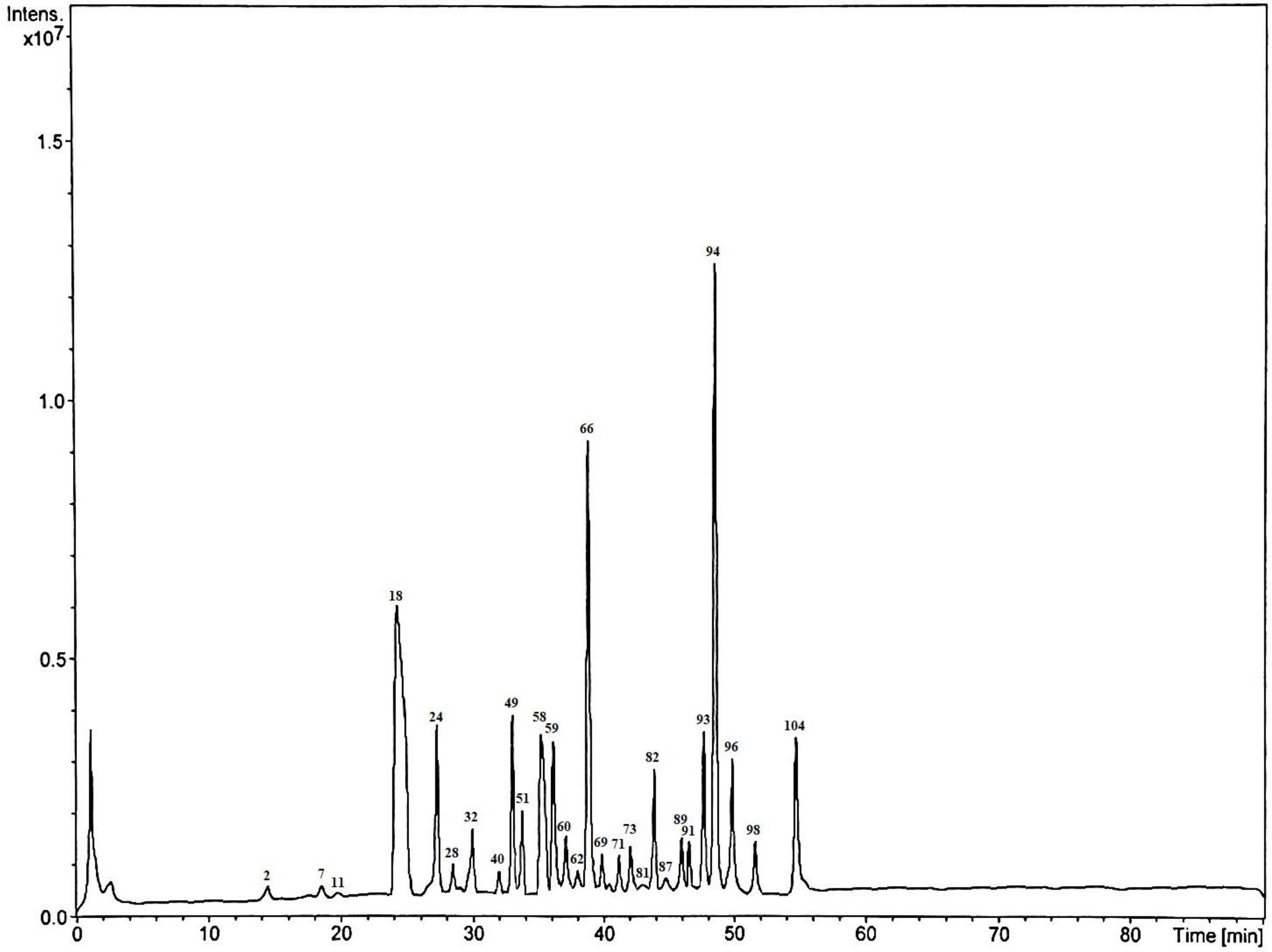

Figure S4. Representative LC-MS chromatogram of *M. pulegium* flower extract I (MPFE I). Peak numbers refer to compounds listed in Table 3.

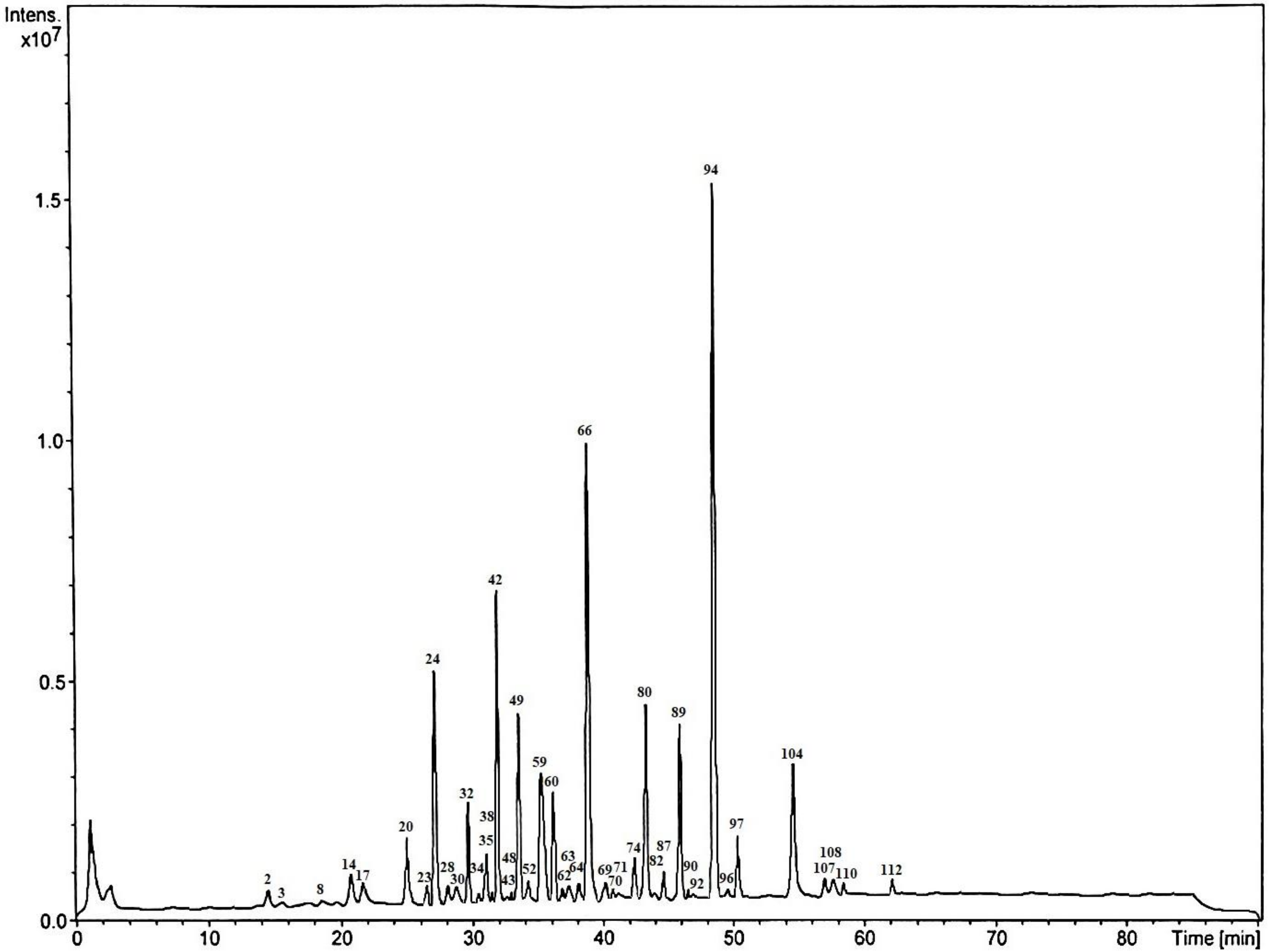

Figure S5. Representative LC-MS chromatogram of *M. pulegium* flower extract II (MPFE II). Peak numbers refer to compounds listed in Table 3.

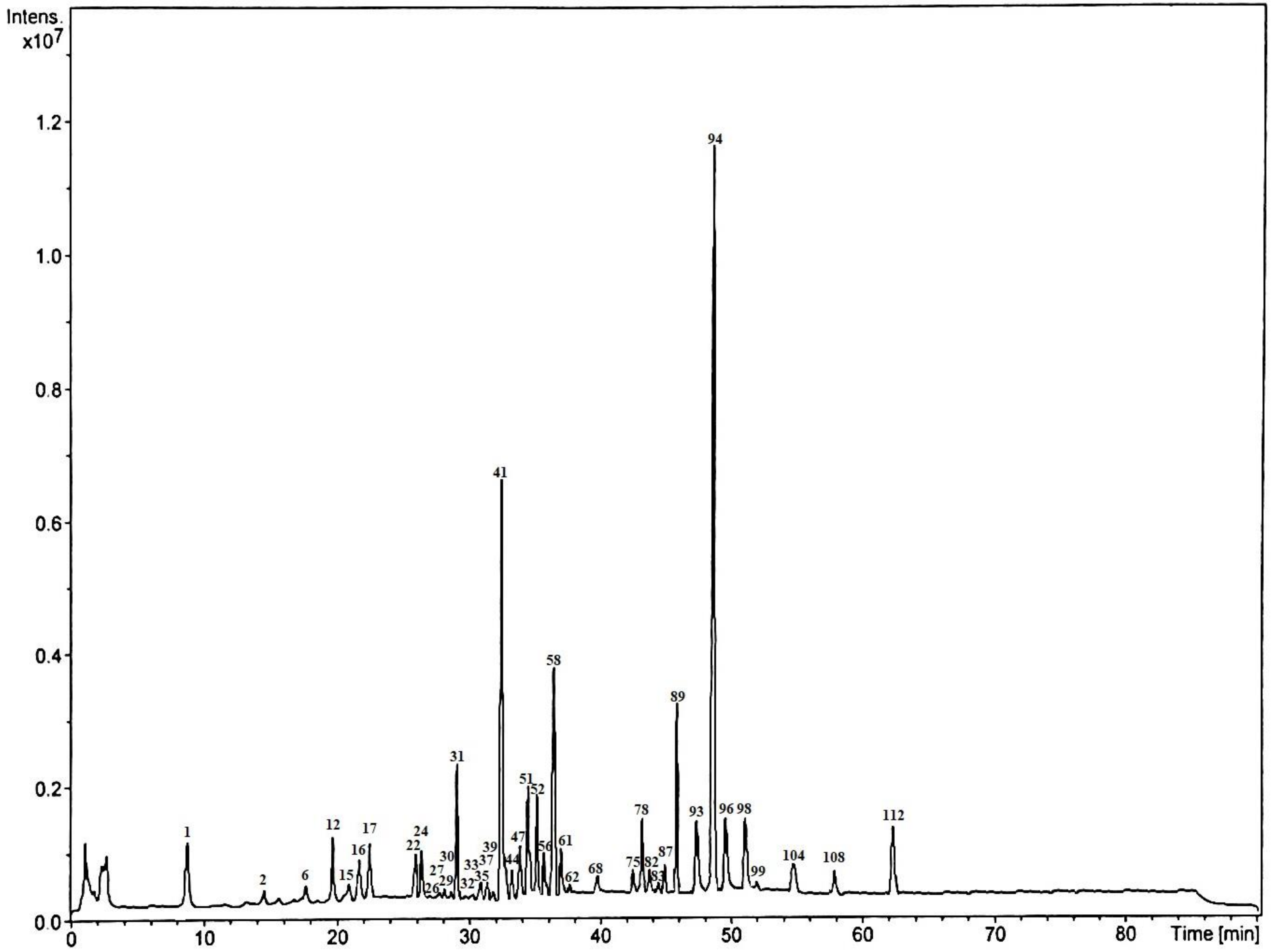

Figure S6. Representative LC-MS chromatogram of *M. pulegium* flower extract III (MPFE III). Peak numbers refer to compounds listed in Table 3.
